# Crossing the Oxo‐Peroxo Wall for Selective Electrochemical Epoxidation

**DOI:** 10.1002/advs.202517229

**Published:** 2025-11-04

**Authors:** Pooja Basera, Shyama Charan Mandal, Frank Abild‐Pedersen, Michal Bajdich

**Affiliations:** ^1^ Department of Chemical Engineering Stanford University Stanford CA 94305 USA; ^2^ SUNCAT Center for Interface Science and Catalysis and Liquid Sunlight Alliance SLAC National Accelerator Laboratory Menlo Park CA 94025 USA

**Keywords:** electrocatalysis, epoxidation, Oxo‐wall, oxygen evolution reaction, peroxo

## Abstract

Electrochemical oxidation in water requires the formation of reactive oxygen species to be able to oxidize unsaturated hydrocarbons to epoxides, aldehydes, and ketones. These reactions, broadly classified as alternative oxidation reactions (AOR), directly compete with the prevalent oxygen evolution reaction (OER). In molecular catalysis, the Oxo‐Wall dictates a transition from a stable oxo intermediate (OER active) to a meta‐stable metal‐oxo (OER inactive) generally occurs. In this work on heterogeneous catalysis, the same Oxo‐Wall applies, however, a meta‐stable oxo preferentially coordinates with lattice oxygen to form a more stable surface peroxo intermediate. A universal free energy onset of this process is identified at 3.39 eV under electrochemical activation in water and show that it is completely decoupled from the OER oxo species. Such decoupling gives rise to a new region of oxygen reactivity relevant for AOR where a selective oxidation of the unsaturated C‐C bonds is predicted to occur instead of OER. A distinct AOR overpotential volcano is constructed and identify recently reported electrocatalysts, including palladium‐platinum for propylene epoxidation and silver‐nickel for ethylene epoxidation, along with others such as TiO_2_ and CuO. Broader implications and limitations of electrochemical AOR are discussed, highlighting their potential to enable electrochemically enhanced thermal catalysis.

## Graphical abstract

The classical Oxo‐Wall concept is extended to an Oxo–Peroxo Wall framework, showing that meta‐stable oxo species favor peroxo and superoxo formation, decoupling from oxygen evolution reaction (OER). This new understanding enables selective C‐C bond oxidations and provides a predictive alternative oxidation reaction (AOR) volcano for designing metal‐oxide catalysts for sustainable electrochemical transformations.

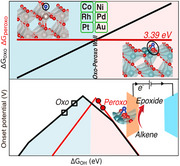

## 1 Introduction

Oxidative electrochemistry is essential for sustainable energy conversion, particularly in advancing the hydrogen economy.^[^ ^1^, ^2^ ^]^ Central to this field is the selective oxidation driven by water‐activation intermediates at the anode, which is instrumental in oxidation catalysis and energy conversion technologies.^[^ ^3^ ^]^ Active oxygen species, such as oxo,^[^ ^4^ ^]^ oxyl,^[^ ^5^ ^]^ and peroxo,^[^ ^6^ ^]^ can be distinguished based on the binding energy of the adsorbed oxygen and its associated bond lengths. Gray and Winkler's Oxo‐wall framework,^[^ ^4^ ^]^ based on multi‐bonded metal‐oxo complexes, systematically delineates the stability of reactive oxygen species (ROS) across transition metals molecular complexes. The Oxo‐Wall is a ligand‐field/molecular orbital (MO) framework developed for molecular complexes that explains why terminal metal‐oxo (M = O) multiple bonds are stabilized for early transition metals but become unfavorable for late metals due to filling of *π* ^*^ antibonding orbitals.^[^ ^7^ ^]^ This trend explains the rarity of late‐metal oxo complexes and the appearance of the alternative O‐species such as peroxo/superoxo's.^[^ ^8^, ^9^ ^]^ Importantly, the ‘Oxo‐Wall’ is not rigid, recent studies indicate that late‐metal oxo/oxyl units are best viewed as a quantum‐mechanical mixture of M^IV^ = O and M^III^–O· rather than pure species.^[^ ^10^, ^11^ ^]^ The concept is central to the oxidation catalysis because its accessibility and longer lifetime of high‐valent M = O intermediates that can govern O‐H/C‐Hactivation and O‐O bond formation in enzymatic and synthetic systems.^[^ ^4^, ^8^ ^]^

Promising alternatives to conventional water oxidation reaction, which do not produce useful fuels, include water oxidation to peroxides,^[^ ^12^ ^]^ light olefin epoxidation,^[^ ^3^ ^]^ and alcohol oxidation.^[^ ^13^ ^]^ Among these alternative oxidation reactions (AOR), the ethylene and propylene epoxidation is of particular interest due to their importance as fundamental building blocks in the chemical industry,^[^ ^14^, ^15^ ^]^ being widely used for manufacturing antifreeze, solvents, pharmaceuticals, textiles, and other functional materials.^[^ ^16^, ^17^ ^]^

Recent advances, such as those by the Manthiram group, have shown that palladium‐platinum (Pd‐Pt) electrocatalysts facilitate water activation via peroxo‐type oxygen intermediates, enabling direct propylene epoxidation to yield propylene oxide.^[^ ^3^ ^]^ Artiglia et al. highlighted the structure and reactivity of active oxygen species on silver (Ag) surfaces for ethylene epoxidation. They observed a tilted dioxygen structure on partially oxidized Ag(111) surfaces with an O‐O bond distance of 1.48 Å, which may potentially indicate a peroxo species.^[^ ^18^ ^]^ Moreover, a recent study by Christopher et al. has shown that nickel (Ni) doping in Ag nanoparticles promotes selective oxidation by activating molecular oxygen without strongly binding it on the catalytic surface, thereby enhancing selectivity for ethylene oxide production.^[^ ^19^ ^]^ Flaherty et al. further demonstrated that epoxidation and oxygen evolution reactions (OER) on gold anodes share a common pool of reactive oxygen atoms but diverge mechanistically.^[^ ^20^ ^]^ Yamamoto and co‐workers have reported enantioselective epoxidation of β,γ‐unsaturated carboxylic acids using a binuclear titanium complex where reactive oxygen is peroxo.^[^ ^21^ ^]^ In general, when oxo is lower in Gibbs free energy than peroxo on a given surface, OER is preferred; if peroxo is lower, epoxidation is preferred by rapidly reacting with the *π*‐electron cloud of the unsaturated hydrocarbons to form a three‐membered cyclic ring^[^ ^22^, ^23^ ^]^ Organic peroxides or peracids have traditionally been used for epoxidation,^[^ ^22^ ^]^ but their hazards have driven interest in greener routes like H_2_O_2_ as a safer peroxo source.^[^ ^24^, ^25^ ^]^ All these reports suggest that the presence of reactive oxygen may open a new direction for AOR.

Additionally, other oxidative transformations have been explored, such as the oxidation of benzyl alcohol to benzoic acid, HMF (5‐Hydroxymethylfurfural) to FDCA (2,5‐furandicarboxylic acid), and others.^[^ ^13^, ^26^, ^27^ ^]^ Theoretical insights reveal the crucial role of direct C‐O bonding via activated surface oxygen or its vacancies in facilitating these reactions. Zhu et al. found that peroxo species tune surface reactivity in CuO for H_2_ and CO oxidation reactions.^[^ ^6^ ^]^ Huang et al. identified both peroxo and superoxo species on ceria (CeO_2_) surfaces at low‐temperature oxidation reactions.^[^ ^28^ ^]^ Jean‐Marie et al. further visualized these species in layered oxides, linking oxygen redox activity to the stability of lattice oxygen in lithium‐ion batteries and Co‐─Zn oxyhydroxide electrocatalysts.^[^ ^29^, ^30^ ^]^

In the above oxidative reactions, ROS such as oxo (O^*^), peroxo (O^*^O), and superoxo (O_2_^*^), play significant roles when metal oxides are present. Understanding the role of ROS in modulating catalytic performance in anodic reactions requires a broader evaluation of oxygen bonding strength across transition metal oxides. While the oxygen evolution reaction is well known for metal‐oxides with strong to moderately bonded oxygen, such as in IrO_2_ and RuO_2_,^[^ ^31^, ^32^ ^]^ chemistry of a weakly bonded metal‐oxides is significantly less explored, despite its big potential in producing value‐added products in place of O_2_ in photoelectrochemical solar‐fuel devices.^[^ ^33^ ^]^

In this study, we start by introducing the atomic and electronic structure of ROS adsorbed on metal oxides to distinguish between oxo, peroxo, and superoxo species. Secondly, we have computationally re‐evaluated a weakly bonded oxo region of the transition metal oxides to identify the change from oxo‐bonded intermediates to peroxo‐bonded ones. Using our conjecture that such reactive oxo's can be stabilized with nearby lattice oxygens in the form of peroxo/super‐oxo with a lifetime long enough to react with unsaturated C‐C bonds, we have constructed a new AOR overpotential volcano distinct from the OER volcano. Lastly, we validated our AOR overpotential volcano against well‐characterized recent experimental systems that demonstrate the potential for peroxo mediated AOR in ethylene or propylene epoxidation and related reactions.

## 2 Results and Discussion

### 2.1 Electronic Structure of Oxo, Peroxo, and Superoxo

**Figure 1:**
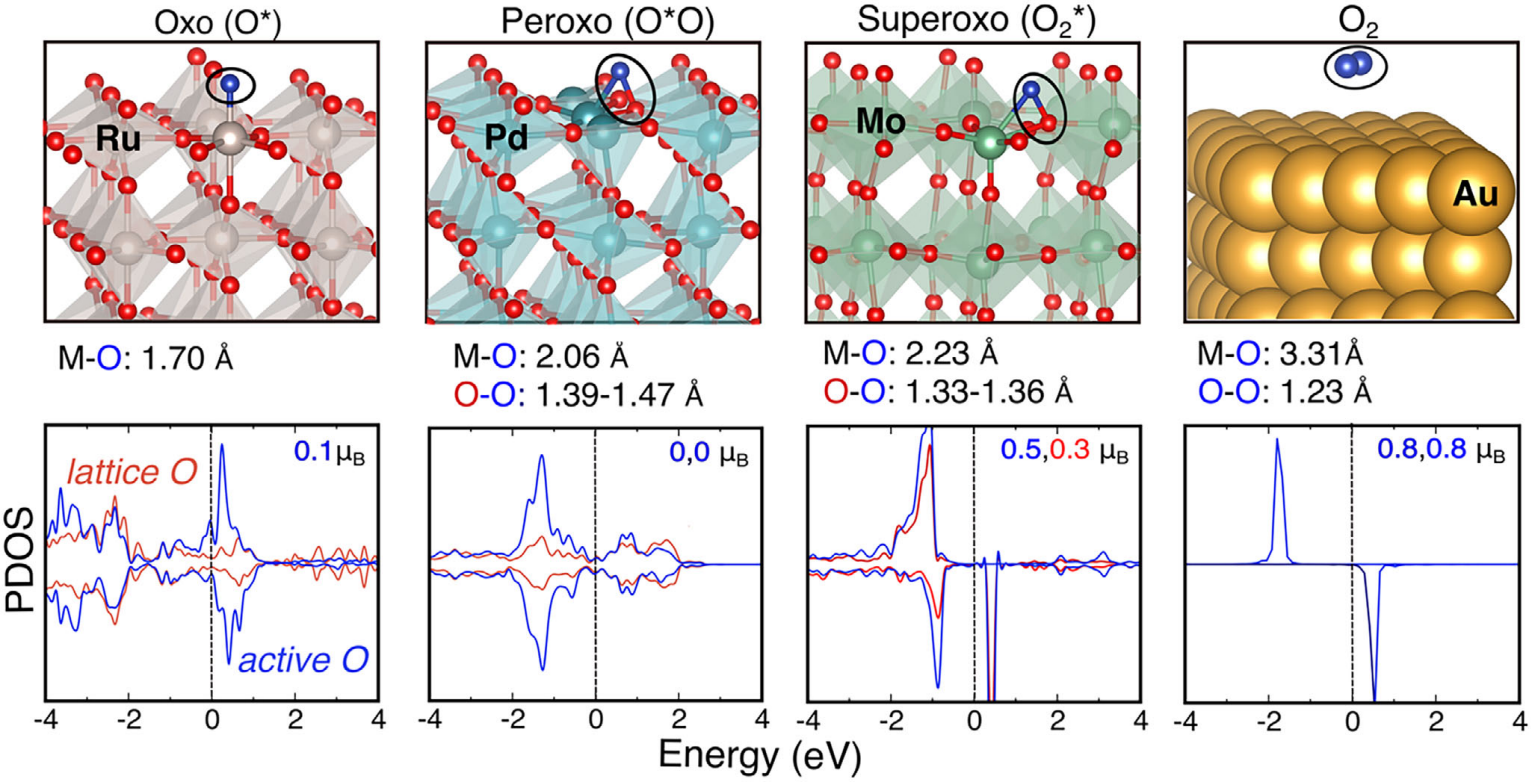
Spectrum of reactive oxygen species observed on metal oxides (and metals). The atomic models represent reactive oxygen species, from most to least interacting; including oxo (O^*^) at top sites [RuO_2_(110)], peroxo (O^*^O) [PdO_2_(110)], superoxo (O_2_^*^) [MoO_3_(001)], and molecular oxygen (O_2_) [Au(111)], along with their respective M(metal)‐O and O‐O bond lengths. The projected DOS for lattice oxygen (red) and active oxygen species (blue), illustrating the electronic interactions between the adsorbed reactive oxygen atom and the lattice oxygen atoms in metal oxides. For summary of the features please refer to **Table** **1**.

A schematic overview of reactive oxygen configurations and their electronic properties are summarized in **Figure** **1**. They span from double M‐bonded oxo (O^*^), lattice oxygen coordinated peroxo (O^*^O), and super‐oxo (O_2_^*^), all the way to molecular adsorbed O_2_. Here, the signatures are extracted from realistic catalytic surfaces of reactive oxygen adsorbed on metal‐oxides [RuO_2_ (110), PdO_2_ (110), MoO_3_ (110)] and metallic gold [Au(111)] surfaces.

An oxo species interacting strongly with the surface exhibits short M‐O^*^ bonds, particularly in metal oxides such as RuO_2_ (M‐O: 1.70 Å), forming multiple highly covalent metal–oxygen bonds. In the following, weak limiting case of oxo with a single M‐O^*^ bond, often called as oxyl^[^ ^5^ ^]^ discussed by Gray and Winkler,^[^ ^4^ ^]^ is not explicitly distinguished from strong oxos for simplicity here.

On the other hand, dual metal and lattice oxygen coordinated oxygens can form peroxo and superoxo species that show much weaker metal‐oxygen bonds on surfaces like PdO_2_/TiO_2_ (110) and MoO_3_ (001), with bond lengths of 2.06 and 2.23 Å, respectively (Figure 1). When these sites are available, atomic oxygen prefers to stabilize by coordinating with a lattice oxygen and a metal site, over forming weak oxo (oxyl). The resulting O^*^‐O(lattice) bond lengths for the peroxo species range from 1.39–1.47 Å and for superoxo species from 1.33–1.36 Å. The limiting case for no M‐O^*^ interaction is illustrated by molecular O_2_ physisorbed on the gold (Au) surface, thus resulting in negligible surface interactions (Figure 1). In general, when moving from peroxo to molecular adsorbed O_2_, the M‐O bond length increases (weakens) while the O‐O bond length decreases (strengthens).

The electronic fingerprints extracted from the projected DOS, ICOHP analysis^[^ ^34^ ^]^ and Bader charges^[^ ^35^, ^36^ ^]^ (summarized in Table 1) reveal that for the oxo intermediate, there is equally strong hybridization between the oxygen and metal as between the lattice oxygen and metal. Here, the spin states are typically fully quenched for oxo (singlet, µ_B_ ≈0) and partially quenched for oxyls ((µ_B_ ≲ 1)). These species also exhibit the most negative Bader charge values of −0.73|e| due to most charge transfer from metal to oxygens. For the peroxo species, the combined spin states of the O^*^‐O*_latt._* pair is typically a singlet, whereas superoxo species typically show spin asymmetry akin to a weak doublet. The physisorbed O_2_ shows discreate states featuring a molecular‐like triplet state. The Bader charges reflect a moderately negative peroxo species (O^*^: −0.45, O*_latt._*: −0.49 |e|), less negative superoxo species (O^*^: −0.17, O*_latt._*: −0.27 |e|), and nearly neutral physisorbed O_2_ (≈−0.02 |e|), having a decreasing degree of electron transfer from the metal to oxygens. The magnetic moments for oxo (O^*^), oxyl (M‐O•), peroxo (O^*^O), and superoxo (O_2_^*^) species on various surfaces plotted in Bohr magnetons (µB) are provided in Figure S8 (Supporting Information). In general, the signatures from metal‐oxides confirm the previous assignment from metallic surfaces.^[^ ^37^, ^38^ ^]^ The next step is to identify catalytic systems which preferentially stabilize peroxo over oxo, and tunability towards higher AOR selectivity.

**Table 1:** Summary of electronic signatures for reactive oxygen species (ROS). Analysis for metal oxide and metal surfaces. The values are in order for active oxygens (O^*^) and lattice oxygens (O*_latt._)* atoms, respectively.

Species: systems	Spin State	Bond order	Magnetic moments [µB] [O^*^/O*_latt._*]	Bader charges |e| [O^*^/O*_latt._*]	Formula/ formal charge
Oxo (O^*^): RuO_2_	quenched	1–3	0.1	−0.73	O^2 −^ /−2
Peroxo (O^*^O): TiO_2_, PdO_2_	singlet	1	0/0, 0/0	−0.45, −0.49/ −0.18, −0.32	$O_{2}^{2-}$/−1
Superoxo (O_2_^*^): MoO_3_	doublet	1.5	0.5/ 0.3	−0.17, −0.27	$O_{2}^{-}$/−0.5
Molecular O_2_	triplet	2	0.8/ 0.8	0.02, −0.02	O_2_/0

### 2.2 Oxo‐Peroxo Wall in the Metal Oxides

The presence of an oxo‐wall proposed by Gray and Winkler in molecular complexes^[^ ^4^ ^]^ suggests that stable metal‐oxo featuring two or more bonds exists only for the first half of the periodic table. Beyond this limit, meta‐stable, singly bonded oxos (i.e., oxyls) can form only in a very high oxidation states (+5 and greater).

Electrochemically, such conditions are present under high applied anodic bias (*U_RHE_* > 1.8 V), at which unstable oxo species rapidly form water‐oxidation products such as O_2_ and/or H_2_O_2_.

**Figure 2:**
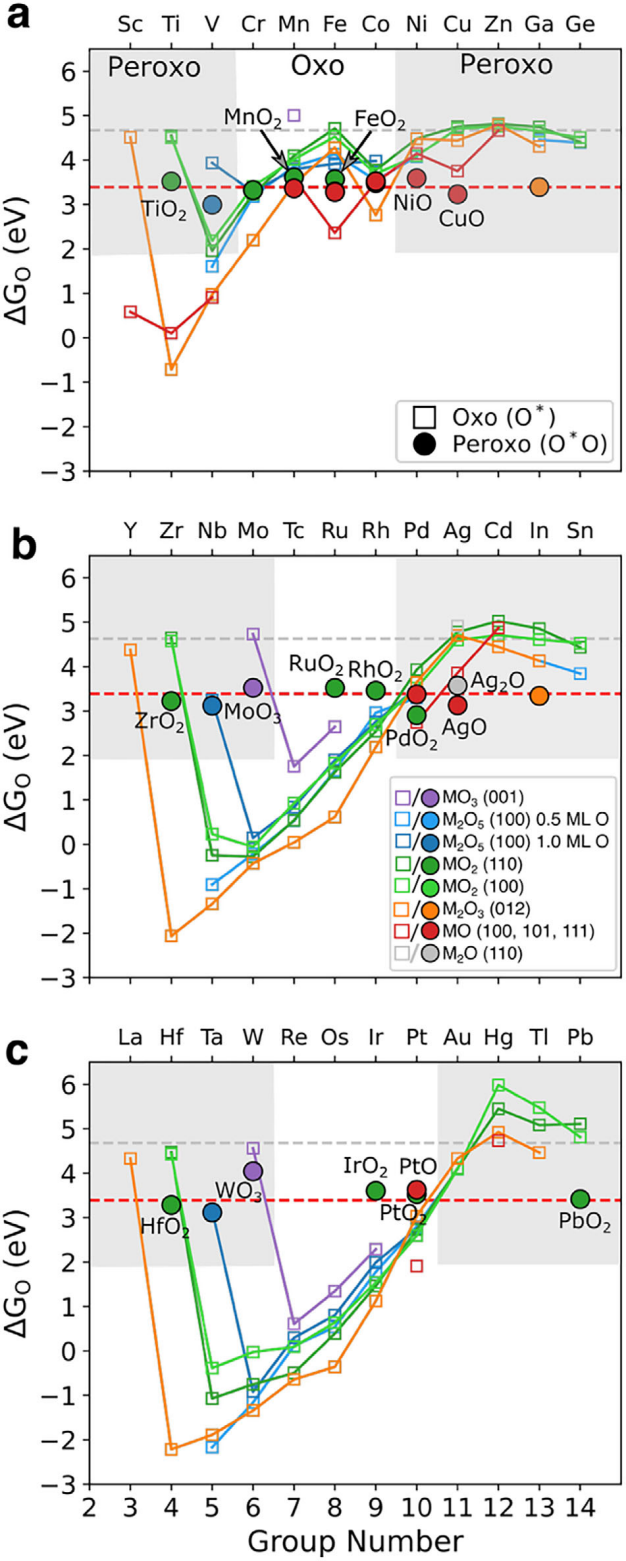
Oxygen adsorption energies across the Oxo‐Peroxo Wall for transition metal‐oxides. We plot a) 3d, b) 4d and c) 5d transition metal oxides in oxidation states from +1 to +6, with oxos (empty symbols) having the V‐shape dependence with group number while peroxo/superoxo (full symbols) featuring flat dependence. The dashed red‐line (3.39 eV) highlights the location of the energetic transition between oxo‐peroxo which coincides with doubly bonded M‐oxo transitions to singly bonded (empty symbols vs full symbols). Each plot has shaded and unshaded regions: the shaded region favors peroxo formation with lattice oxygen, while the unshaded favors oxo. The boundary defines the Oxo–Peroxo Wall. Weak bonding limit of singly bonded oxygen shown as a dashed grey line (4.65 eV). Light blue denotes 0.5 ML O‐atom coverage, and dark blue denotes 1.0 ML O‐atom coverage (ML = monolayer).

The depiction introduced by the oxo‐wall picture is very closely aligned with our recent survey of O^*^ and OH^*^ adsorption energies across the surfaces of transition metal‐oxide series in octahedral coordination^[^ ^39^ ^]^ (**Figure** **2**, empty symbols). The observed V‐shape adsorption behavior of the on‐top adsorbed oxygen (oxos) energies across the periodic table (empty symbols) suggests that at the global adsorption minimum (strongest) is observed for d^1^ (group 3 for MO, group 4 for M_2_O_3_, group 5 for MO_2_, etc) and steadily weakens with increasing number of anti‐bonding states. Systems with d^0^ (empty), d^10^ (fully‐filled), and d^5^ (half‐filled high‐spin) bulk metal‐oxide occupations feature a significantly weakened adsorption (i.e., TiO_2_, V_2_O_5_, In_2_O_3_, PbO_2_ etc). In our earlier study,^[^ ^39^ ^]^ we defined a weakly bonding limit at 4.65 eV (vs H_2_O*(l)*/H_2_) as the adsorption limit of oxo's for d^0^/d^10^ bulk systems. These on‐top adsorption energies have been extracted from the most common facets of the rocksalt MO (100), corundum M_2_O_3_ (012), rutile MO_2_ (110), M_2_O_5_ (100), and MO_3_ (001) highly oxidized bulk structures, with full details in Ref. [39]. In this study, we additionally included stable square planar CuO (111), AgO (111), and tetrahedral PdO (101), and PtO(101) surfaces,^[^ ^6^, ^40^, ^41^ ^]^ as well as Ag_2_O (110).

Increasing number of anti‐bonding states leads to a decrease of the oxo's stability; in some cases, a weakly coordinated oxos (oxyls) were unstable and preferred to coordinate partially with oxygen. Hence, in nearly all cases, we can form a very stable peroxo/super‐oxo intermediate in these systems (full symbols, Figure 2). Strikingly, the adsorption energy of such oxygens remains constant at 3.39 eV (red dashed line), which is below the d^0^, d^10^, and d^5^ saturated oxo line 4.65 eV (grey dashed line).

The energetic trend shown in Figure 2 highlights three distinct regions based on the relative stability of oxo and peroxo species. The shaded regions indicate where the peroxo species is thermodynamically favored over oxos, clearly defining an Oxo–Peroxo Wall that divides the 3d, 4d, and 5d metal oxides into oxo‐preferred (unshaded) and peroxo‐preferred (shaded) regions. On the right side, this crossover aligns with the oxo‐wall for molecular complexes,^[^ ^4^ ^]^ occurring between Co‐Ni (3d), Rh‐Pd (4d), and Pt‐Au (5d). On the far left, stable multiple‐bonded oxo species dominate, however, only higher oxidation states such as +4 (TiO_2_), +5 (V_2_O_5_), and +6 (MoO_3_) form more preferred peroxo or superoxo intermediates than oxos. This indicates that the left position of the Oxo‐Peroxo Wall moves depending on the oxidation state. In the middle region, oxo species are generally the more preferred, as is evidenced for 4d and 5d metal oxides such as RuO_2_, RhO_2_, IrO_2_, PtO_2_, PtO etc. For 3d metal oxides, few exceptions, such as FeO_2_ (110) and MnO_2_ (110) feature less preferred oxo (more preferred peroxo) due to saturated high spin d^5^ configurations.

### 2.3 Decoupling of Peroxo from Oxo Species and New Oxidative Volcano Relationship

**Figure 3:**
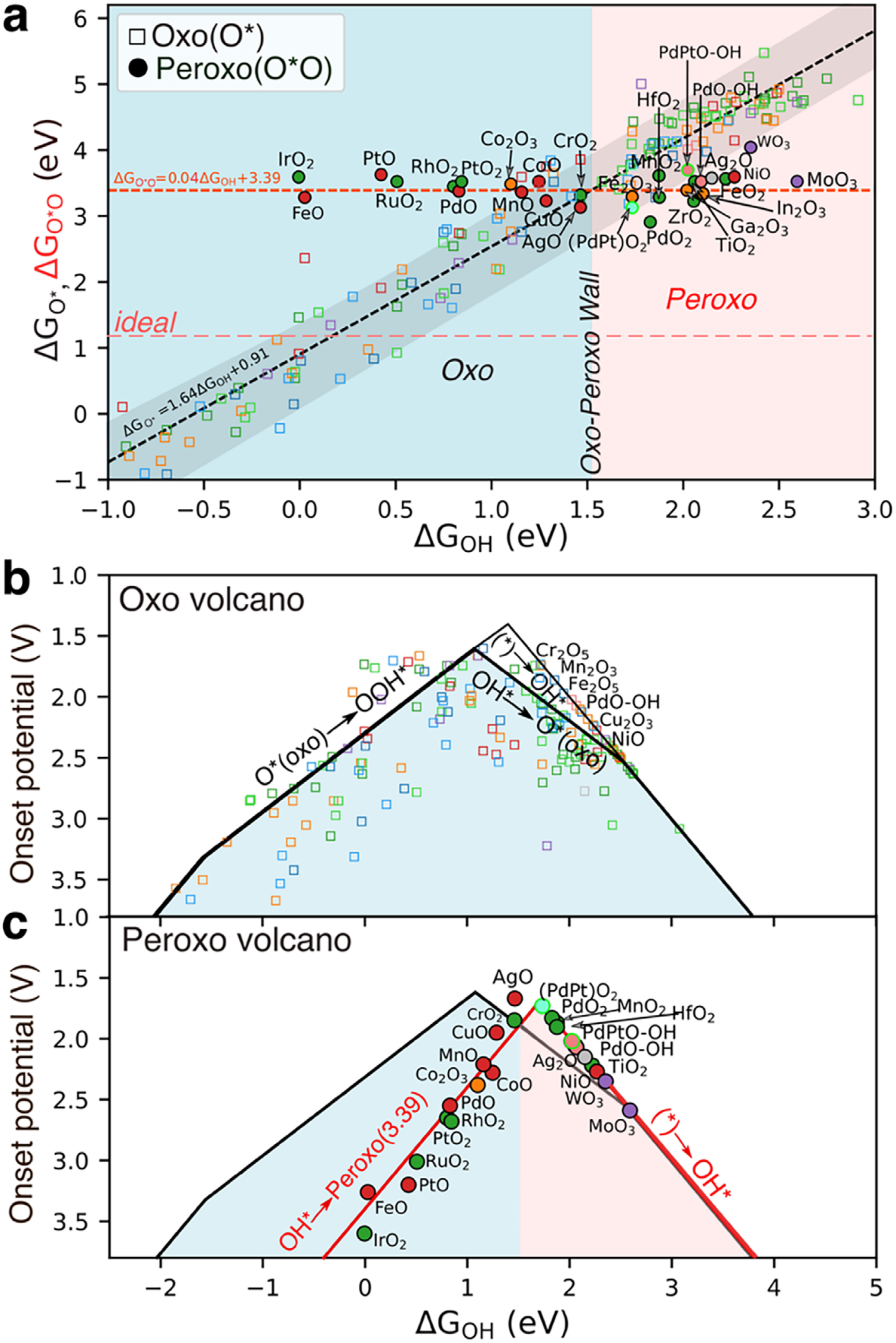
Adsorption scaling relations for oxo and peroxo surface adsorbates, and associated oxo and peroxo OER‐type volcanoes. The singly M coordinated O^*^ (oxo's) with OH^*^ (black line) and surface coordinated peroxo (red line) for the subset of data from Figure 2. The oxo (O^*^) follows a typical dependence on OH^*^ with slope ≈1.64, and ≈1.2 for 3d metals. Peroxo (O^**^O, red line) remains nearly constant at ≈3.39 eV; the dashed light red line marks the ideal peroxo free energy (1.2 eV). The Oxo–Peroxo Wall occurs at a critical OH^*^ value of 1.55 ± 0.1 eV. Blue shading marks the oxo‐favorable region; red and mixed shading indicate peroxo‐favorable zones. b) The OER volcano, shaded in blue, is constructed using the free energy steps of a single‐site OER mechanism and the scaling relation from panel a). The marked squares denote the OER‐active species. c) Peroxo volcano (red lines) derived from (a), with splitting at OH^*^ energy of ≈1.55 eV; blue, transition from blue to red, and red areas indicate OER, Oxo‐Peroxo Wall, and peroxo‐active regions, respectively.

Next, we examine how the formation of the stable peroxo (O^*^O) species compares to oxo (O^*^) species on the relative scale set by OH^*^ adsorption as shown in **Figure** **3**a. Since OH^*^ acts as a precursor to activated oxo as well as peroxo, it serves as a common descriptor for both intermediates.

A well‐known linear scaling relationships between O^*^(oxo) vs OH^*^ in metal‐oxides^[^ ^39^ ^]^ can be expressed as ΔG_O*_ = *a* ΔG_OH_ + *b* with slope *a* ≈1.64 (and ≈1.3 for 3d metals^[^ ^42^ ^]^), and an intercept of *b* = 0.91 eV. While approximate, especially due to spin effects in 3d metal‐oxides, this scaling linearly depends on M‐O bonding with a bond order lower than 2. This is dramatically different from the peroxo vs OH^*^ scaling (ΔG_O*O_ = 0.04 ΔG_OH_ + 3.39 eV), which is nearly independent of OH^*^ and has a high intercept of 3.39 eV. This observation implies that peroxo maintains an equal sum of M‐O and O‐O bonding and its independent of M‐O bonding. Fundamentally, this also implies that any oxo (O^*^) bonded weaker than this value, ΔG_O_ = +3.39 eV vs H_2_O(l)‐H_2_(g) is unlikely to remain singly bonded to the metal. Instead, it will prefer to coordinate as a peroxo species if lattice oxygen is available. The crossover value for OH^*^ at the oxo‐peroxo wall on the scale can be assigned at ≈1.55(±0.1) eV, marking the point where M‐O bonding weakens, and O‐O bonding becomes stronger. Note that the peroxo relation ΔG_O*O_ = 0.04 ΔG_OH_ + 3.39 eV is a DFT‐based empirical scaling, analogous to the well‐known OER scaling ΔG_OOH_ = ΔG_OH_ + 3.2 eV, which is likewise empirically fitted based on DFT values across many oxide surfaces.^[^ ^43^, ^44^ ^]^

In Figure 3a, in addition to octahedral surfaces from our previous work,^[^ ^39^ ^]^ we also computed adsorption energies for catalytic systems (PdO‐OH, PdO_2_, (PdPt)O_2_, AgO, Ag_2_O, NiO, CuO, TiO_2,_ etc.), where the identity of the intermediate ROS species (oxo, peroxo, or superoxo) in reaction mechanisms has been experimentally studied recently.^[^ ^3^, ^6^, ^18^, ^19^, ^45^, ^46^ ^]^ Notably, we found that most of these recent systems lie on the weak OH^*^ side after the oxo‐peroxo crossover. Based on the reaction free energy analysis, ΔG_O*O_ < ΔG_O*_ it indicates that the ROS intermediates in these systems are more likely to be peroxo species. Such points also adhere well to the new scaling relation observed for peroxo species, further supporting the general validity of our analysis.

In contrast, systems such as IrO_2_, FeO, PtO, PtO_2_, RuO_2_, and RhO_2_ in Figure 3a lie on the strong bonding OH^*^ side. Their oxo species are more preferred than the peroxo species (ΔG_O*O_ > ΔG_O*_) due to stronger M‐O bonds, which leads to fast OER or surface oxidation, although their peroxo species still follow the general trend of 3.39 eV. This finding is consistent with the literature, where these systems are well known for their role in oxygen evolution reactions (OER).^[^ ^31^, ^47^, ^48^, ^49^ ^]^ The trend is also consistent with the Gray and Winkler Oxo‐Wall,^[^ ^4^ ^]^ which places Co, Rh, and Pt on the left and Ni, Pd, Au, Cu, and Ag on the right of their wall (see Figure 2).

We note that PdO (101) typically prefers oxo species. However, the computed surface Pourbaix diagram (see Figure S1, Supporting Information) suggests significant OH coverage under operating conditions, and the resulting 50% OH‐covered PdO surface confirms the stabilization of peroxo over oxo species. Additionally, peroxo formation in d⁰ (corundum structures^[^ ^39^, ^50^ ^]^ of Sc_2_O_3_, Y_2_O_3_, La_2_O_3_, Au_2_O_3_) deviates slightly from ΔG_O*O_ = 0.04 ΔG_OH_ + 3.39 eV, due to polymorph instability (see Figure S7, Supporting Information) which leads to local structural distortions.^[^ ^50^ ^]^ Overall, we identify a universal free energy onset of this process at 3.39 eV under electrochemical activation in water. Such decoupling gives rise to a peroxo volcano distinct from the OER volcano to elucidate the selectivity between oxo and peroxo species.

As a benchmark, we first examine our energetics in the well‐known OER volcano^[^ ^51^, ^52^ ^]^ (Figure 3b), based on the oxo scaling relation (ΔG_O_ = 1.64ΔG_OH_ + 0.91) and OOH^*^ scaling (ΔG_OOH_ = ΔG_OH_ + 3.2) relation. This yields a volcano‐shaped correlation between catalytic activity and OH^*^ Gibbs free energy, with potential limited by either OOH^*^ formation for strong O^*^/OH^*^ binding or OH^*^ oxidation for weak oxygen binding. The theoretical overpotential is given by, η^OER^ = (max [ΔG_OH_, ΔG_O_ – ΔG_OH_, ΔG_OOH_ – ΔG_O_, 4.92 – ΔG_OOH_]/e) −1.23 V. The shaded region in Figure 3b highlights the OER‐active region, with squares indicating the OER‐active species. Lastly, our theory also predicts a few systems which have very unstable oxo, that are limited due to (^*^) + H_2_O → OH^*^ onset, but are experimentally inaccessible (i.e., Cr_2_O_5_, Fe_2_O_5_, Cu_2_O_3_) and should be ignored.

Lastly, on top of the oxo volcano, we can now construct a distinct peroxo volcano shown in Figure 3c, which highlights the selectivity window between oxo and peroxo pathways to be discussed next. The peroxo volcano (solid red line) is constructed using ΔG_1_ = ΔG_OH_, ΔG_2_ = ΔG_O*O_ – ΔG_OH_, with ΔG_O*O_ = + 3.39 eV (setting the slope to zero) as derived from Figure 3a. Directly, we can see that surfaces that bind O^*^/OH^*^ too strongly are limited by peroxo formation, while those with weak binding are limited by OH^*^ formation (as in Figure 3c). The maximum potential‐determining step for peroxo intermediates is given by *max*G^O*O^ = max [ΔG_1_, ΔG_2_]. As a result, a secondary peroxo volcano (solid red line) upshifted from the OER volcano is formed with a red shaded region indicating preferable peroxo formation. Below the Oxo‐Peroxo Wall onset (1.55(±0.1) eV), the OER is still preferred (blue region). Mostly d^0^ and d^10^ system with weakly bonded OH^*^/O^*^ are situated on the weak leg, with some overlap into the strong leg. Notably, superoxo intermediates (e.g., on MoO_3_ and AgO surfaces) are implicitly captured in the peroxo side of the volcano plot and can indeed contribute to oxidative reactivity. 2D heat maps for OER and peroxo, and the 1D volcano plot, which uses ΔG_O_ – ΔG_OH_ as the descriptor, are shown in, Figures S3–S6 (Supporting Information). Overall, the decoupling between oxo and peroxo volcanoes defines a new selective window relevant for AOR, where we predict the selective oxidation of unsaturated C‐C bonds, such as epoxidation and ketonization reactions, to occur instead of OER as we discuss next.

### 2.4 Selectivity Volcano Plot and Epoxidation of 1‐Propylene By Peroxo Species

**Figure 4:**
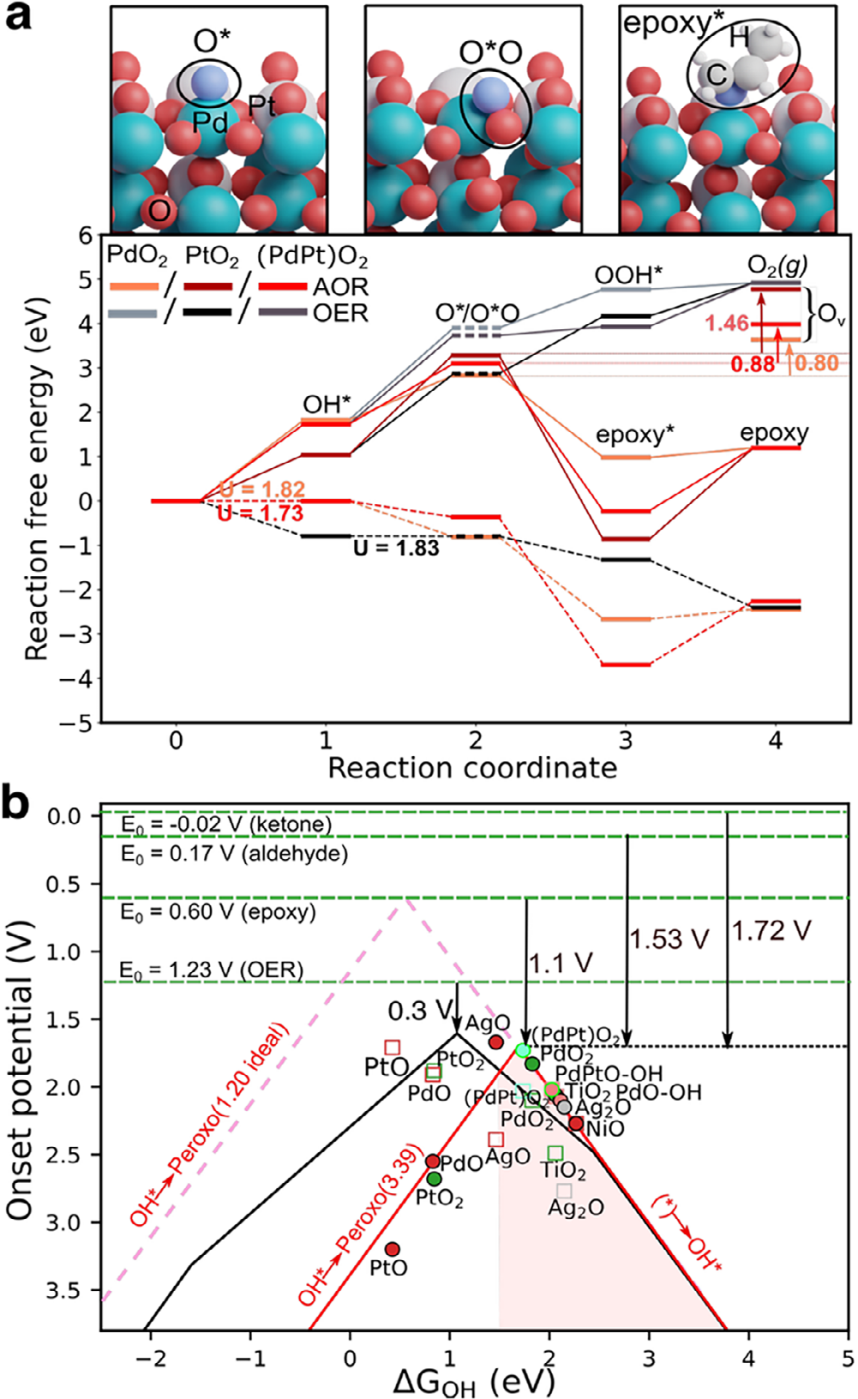
Selectivity and Activity trends between AOR and OER. a) The OER and epoxidation reaction free energy profiles of PdO_2_, PtO_2,_ and (PdPt)O_2_ with and without applied potential. PtO_2_ prefers OER, whereas PdO_2_ and (PdPt)O_2_ prefers epoxidation due to the relative oxo vs. peroxo energies. The inset shows the associated optimized structures for the oxo, peroxo, and epoxy on (PdPt)O_2_ surface. The dashed black/grey line for the oxo species (O^*^) at the second step is used to distinguish from peroxo species (O^*^O). b) Combined oxo (black) and peroxo (red) volcano plots. The oxo (OER) volcano has an overpotential of 0.3 V and an equilibrium potential (E_0_) of 1.23 V RHE. We highlight systems that are well‐established in the literature for epoxidation pathways, where peroxo as the reactive intermediate species. The calculated E_0_ at a propylene concentration of 0.01 M for the epoxidation, aldehyde, and ketonization reactions are 0.6, 0.17, and −0.02 V, respectively. Corresponding calculated minimal overpotentials are 1.1, 1.53, and 1.72 V.

Our calculated results based on the oxo vs peroxo stability open a new direction of epoxidation reaction without the need for hazardous peroxides. As a proof of concept, we have considered propene as a type of an unsaturated hydrocarbon in AOR type reaction for the epoxidation of alkanes. OER and epoxidation routes have been tested for PdO_2_, (PdPt)O_2,_ and PtO_2_ (110) surface (**Figure** **4**a) based on a previous experimental study that used acetonitrile and water as electrolytes.^[^ ^3^ ^]^ In acetonitrile, palladium remains in the +2‐oxidation state, while in water, it may be oxidized to PdO_2_. In our calculations, based on the oxo and peroxo energies it is expected that PdO_2_ will prefer peroxo, i.e., epoxidation reaction, whereas PtO_2_ will favor oxo, i.e., OER. It is noteworthy to mention that the epoxidation is a 2e^−^ oxidation process, whereas OER is 4e^−^ oxidation process, and both the reactions proceed via an OH^*^‐based intermediate.

Figure 4a starts with the calculated reaction free energies of ^*^OH adsorption, which are 1.82, 1.04, and 1.73 eV on PdO_2_, PtO_2,_ and (PdPt)O_2_ (110) surfaces, respectively. The formation of peroxo is −1.07 and −0.63 eV preferred compared to oxo on PdO_2_(110) and (PdPt)O_2_(110) surface, whereas formation of oxo is −0.45 eV preferable compared to peroxo on PtO_2_(110) surface. In the next step, the preferred oxo of PtO_2_(110) can electro‐chemically form OOH^*^ followed by O_2_(g) removal. Crucially, a more preferred peroxo over oxo on PdO_2_ (110) and (PdPt)O_2_(110) surfaces will interact with the unsaturated C═C bond of propene and form propylene oxide. This step is −1.85 and −3.33 eV exergonic (downhill) in nature for PdO_2_(110) and (PdPt)O_2_(110), respectively. It is noteworthy to mention that the OH^*^ and O^*^O formation steps are electrochemically controlled, whereas the formation and removal of epoxide is thermally controlled. We also tested the formation of peroxo‐like OOH^*^ from oxo and/or peroxo and found it highly unfavored and to quickly reverting to singly coordinated OOH^*^ under relaxation. For completeness, we have also computed the generation of O_2_(g) directly from peroxo by forming a lattice vacancy (upward arrows) revealing the unfavourability of direct O_2_ formation from peroxo over epoxidation by at least 0.8 eV.

The required applied potentials to make all the steps downhill and/or reversible process for epoxidation reactions are 1.82, 2.27, and 1.73 V for PdO_2_, PtO_2,_ and (PdPt)O_2_, respectively (lower part of Figure 4a). Besides, the applied potential for OER reactions are 2.08, 1.83, and 2.00 V for PdO_2_, PtO_2,_ and (PdPt)O_2_, respectively. Hence, PdO_2_ and (PdPt)O_2_ will prefer epoxidation, and PtO_2_ prefer OER. Taken together, these results support the epoxidation reaction in the presence of preferred peroxo over oxo and vice versa.

Using the peroxo volcano of Figure 3c, we can now rank a recently reported catalysts^[^ ^3^, ^6^, ^18^, ^19^, ^45^ ^]^ for efficient ethylene and propylene epoxide production and related oxidation reactions, in Figure 4b. Using the experimentally relevant concentrations, we have estimated the equilibrium (or redox) potentials for epoxidation, ketonization, and aldehyde formation reactions to be E_0_ = 0.6, −0.021, and 0.168 V (see Figure S2, Supporting Information). The minimum theoretical overpotentials measured relative to these values as η^*AOR*^ = (*max*G^O*O^/e) – E_0_ correspond to 1.1, 1.72, and 1.53 V, respectively. While not discussed in detail here ketonization and aldehyde formation require extra deprotonation steps which can also be rate limiting in addition to the dominant peroxo formation step.

Nearly all the reported systems (AgO, Ag_2_O PdO_2_, (PdPt)O_2_, PdO‐OH, and TiO_2_) are located on the weak leg of the peroxo volcano and exhibit selectivity for peroxo as a weakly bound ROS in epoxidation reactions. Their onset potentials are near or below 1.7 V with the best catalyst being (PdPt)O_2_ and AgO with experimental values all reported at ≈2 V range.^[^ ^3^ ^]^ While the ideal catalyst could in principle facilitate epoxidation reaction just above the 0.6 V equilibrium potential, such values limited by the ΔG_O*O_ = 3.39 eV scaling from OH*, which would need to be 1.2 eV for an ideal catalyst.

## 3 Conclusion

In summary, our findings extend the classical Oxo‐Wall concept from molecular catalysis to a broader Oxo–Peroxo Wall framework for heterogeneous catalysis. It reveals that beyond this boundary, meta‐stable oxo (oxyls) species favor the formation of stable surface peroxo and superoxo intermediates. This decoupling from the conventional OER pathway defines a new window of oxygen reactivity that enables selective AOR processes. By establishing a universal free energy onset and constructing a distinct AOR volcano, we provide a predictive framework for designing and ranking metal‐oxide electrocatalysts for selective unsaturated C‐C bond oxidation, such as epoxidation and ketonization. This work therefore opens new avenues for electrochemical pathways as sustainable alternatives coupled with thermal oxidation, paving the way for more efficient and selective organic transformations in aqueous media. Future detailed mechanistic studies and experimental validation of additional catalysts predicted by our new volcano and ROS framework for alternative oxidation reactions, such as epoxidation, ketonization, and aldehyde formation will open an exciting direction for future research.

## 4 Experimental Section

Density functional theory calculations were performed using the Perdew–Burke–Ernzerhof (PBE) exchange‐correlation functional to account for exchange and correlation effects with Hubbard‐U^[^ ^53^, ^54^ ^]^ correction, as implemented within the Vienna Ab‐initio Simulation Package (VASP)^[^ ^55^ ^]^ and the atomic simulation environment (ASE).^[^ ^56^ ^]^ Hubbard‐U were used in the metals in the metal‐oxide systems as summarized in the Table S1 (Supporting Information). Projector‐augmented wave potentials were employed to replace the inner cores of the atoms with all‐electron, frozen cores. A plane wave cutoff of 500 eV was selected, and a k‐point density of at least 25 Å was used for surface and bulk systems. The Perdew‐Burke‐Ernzerhof (PBE) exchange‐correlation functional was used to account for exchange and correlation effects.^[^ ^57^ ^]^ The calculations were carried out on a Monkhorst‐Pack grid centered around the Γ‐point. The energy convergence threshold for the relaxation of energies was set to 10^−5^ eV, ensuring the accuracy of the results. Furthermore, atomic forces were converged to 0.01 eV Å^−1^ to ensure stable atomic positions. To ensure accurate enthalpy calculations, we included both zero‐point energies (ZPE) and phonon vibrational energies (U_vib_) into DFT electronic energies. ZPE and U_vib_ calculations were carried out using the independent harmonic oscillator approximation with the ASE Thermochemistry class.^[^ ^58^ ^]^ Notably, the components of free energies and vibration corrections for peroxo (O^*^O*_latt.)_* and active oxygen (O^*^) in the peroxo species are given in Tables S2 and S3 (Supporting Information). To address known inaccuracies in DFT, particularly the over binding of O_2_ and self‐interaction errors, we applied a correction scheme.^[^ ^59^ ^]^ Our approach involves aligning the energy of O_2_ with the experimental formation enthalpy of water, consistent with established methods.^[^ ^60^ ^]^ The adsorption energy of this oxygen is calculated with respect to liquid H_2_O and H_2_. The methodology for calculating the OER overpotential is given in Supporting Information.

## Conflict of Interest

The authors declare no conflict of interest.

## Author Contributions

The manuscript was written through contributions of all authors. All authors have given approval to the final version of the manuscript.

## Supporting information



Supporting Information

## Data Availability

The full list of adsorption total energies are presented in Table S4 (Supporting Information), and their respective energies with optimized structures available at Catalysis‐hub.org^[^ ^61^ ^]^ under https://www.catalysis‐hub.org/publications/BaseraImplications2025.

## References

[1] Z. W. Seh , J. Kibsgaard , C. F. Dickens , I. Chorkendorff , J. K. Nørskov , T. F. Jaramillo , Science 2017, 355, aad4998. 10.1126/science.aad4998 28082532

[2] P. Basera , Y. Zhao , A. T. Garcia‐Esparza , F. Babbe , N. Bothra , J. Vinson , D. Sokaras , J. Yano , S. W. Boettcher , M. Bajdich , J. Am. Chem. Soc. 2025, 147, 16070. 40311110 10.1021/jacs.4c18147

[3] M. Chung , J. H. Maalouf , J. S. Adams , C. Jiang , Y. Román‐Leshkov , K. Manthiram , Science 2024, 383, 49. 38175873 10.1126/science.adh4355

[4] H. B. Gray , J. R. Winkler , Acc. Chem. Res. 2018, 51, 1850. 30016077 10.1021/acs.accounts.8b00245 PMC6106048

[5] Y. Shimoyama , T. Kojima , Inorg. Chem. 2019, 58, 9517. 31304743 10.1021/acs.inorgchem.8b03459

[6] Y. Zhu , J. Wang , S. B. Patel , C. Li , A. R. Head , J. A. Boscoboinik , G. Zhou , Proc. Natl. Acad. Sci. USA 2023, 120, 2215189120. 10.1073/pnas.2215189120 PMC10068848 36943886

[7] V. A. Larson , B. Battistella , K. Ray , N. Lehnert , W. Nam , Nat. Rev. Chem. 2020, 4, 404. 37127969 10.1038/s41570-020-0197-9

[8] E. Andris , R. Navrátil , J. Jašík , M. Srnec , M. Rodríguez , M. Costas , J. Roithová , Angew. Chem., Int. Ed. 2019, 58, 9619. 10.1002/anie.201904546 PMC6618258 31083766

[9] C. Limberg , Angew. Chem., Int. Ed. 2009, 48, 2270. 10.1002/anie.200805977 19191356

[10] A. Sen , G. Rajaraman , Faraday Discuss. 2022, 234, 175. 35147623 10.1039/d1fd00072a

[11] A. Sen , S. Sharma , G. Rajaraman , Angew. Chem., Int. Ed. 2025, 64, 202419953. 10.1002/anie.202419953 39980408

[12] A. Kulkarni , S. Siahrostami , A. Patel , J. K. Nørskov , Chem. Rev. 2018, 118, 2302. 29405702 10.1021/acs.chemrev.7b00488

[13] L. Wei , D. Hossain , M. J. Boyd , J. Aviles‐Acosta , M. E. Kreider , A. C. Nielander , M. B. Stevens , T. F. Jaramillo , M. Bajdich , C. Hahn , ACS Catal. 2023, 13, 4272.

[14] C. Stegelmann , N. C. Schiødt , C. T. Campbell , P. Stoltze , J. Catal. 2004, 221, 630.

[15] N. Kapil , T. Weissenberger , F. Cardinale , P. Trogadas , T. A. Nijhuis , M. M. Nigra , M. Coppens , Angew. Chem. 2021, 133, 18333. 10.1002/anie.202104952 PMC8456944 34085370

[16] G. Sienel , R. Rieth , K. T. Rowbottom , Ullmann's Encyclopedia of Industrial Chemistry, Wiley, Hoboken, NJ, US 2000.

[17] H. Baer , M. Bergamo , A. Forlin , L. H. Pottenger , J. Lindner , Ullmann's Encyclopedia of Industrial Chemistry, Wiley, Hoboken, NJ, US 2012.

[18] M. Guo , N. Dongfang , M. Iannuzzi , J. A. Van Bokhoven , L. Artiglia , ACS Catal. 2024, 14, 10234. 38988650 10.1021/acscatal.4c01566 PMC11232021

[19] A. Jalil , E. E. Happel , L. Cramer , A. Hunt , A. S. Hoffman , I. Waluyo , M. M. Montemore , P. Christopher , E. C. H. Sykes , Science 2025, 387, 869. 39977491 10.1126/science.adt1213

[20] R. Ghosh , G. M. Hopping , J. W. Lu , D. W. Hollyfield , D. W. Flaherty , J. Am. Chem. Soc. 2025, 147, 1482. 39661713 10.1021/jacs.4c08948 PMC11744761

[21] T. Sawano , H. Yamamoto , ACS Catal. 2019, 9, 3384.

[22] K. A. Joergensen , Chem. Rev. 1989, 89, 431.

[23] Q. Wang , Y. Deng , D. Viera , X. Liu , N. Liu , Y. Hu , X. Hu , H. Wei , Q. Zhou , T. Lan , W. He , X. Chen , C. Kim , Angew. Chem., Int. Ed. 2025, 64, 202504982. 10.1002/anie.202504982 PMC12144862 40199722

[24] K. Kamata , K. Yonehara , Y. Sumida , K. Yamaguchi , S. Hikichi , N. Mizuno , Science 2003, 300, 964. 12738860 10.1126/science.1083176

[25] E. Z. Ayla , D. S. Potts , D. T. Bregante , D. W. Flaherty , ACS Catal. 2021, 11, 139.

[26] L. Chico‐Mesa , A. Rodes , R. M. Arán‐Ais , E. Herrero , Nat. Commun. 2025, 16, 3349. 40204756 10.1038/s41467-025-58696-4 PMC11982258

[27] W. R. Birmingham , A. Toftgaard Pedersen , M. D Gomes , M. Bøje Madsen , M. Breuer , J. M. Woodley , N. J. Turner , Nat. Commun. 2021, 12, 4946. 34400632 10.1038/s41467-021-25034-3 PMC8367993

[28] M. Huang , S. Fabris , Phys. Rev. B 2007, 75, 081404.

[29] E. McCalla , A. M. Abakumov , M. Saubanère , D. Foix , E. J. Berg , G. Rousse , M.‐L. Doublet , D. Gonbeau , P. Novák , G. Van Tendeloo , R. Dominko , J.‐M. Tarascon , Science 2015, 350, 1516. 26680196 10.1126/science.aac8260

[30] Z.‐F. Huang , J. Song , Y. Du , S. Xi , S. Dou , J. M. V. Nsanzimana , C. Wang , Z. J. Xu , X. Wang , Nat. Energy 2019, 4, 329.

[31] L. C. Seitz , C. F. Dickens , K. Nishio , Y. Hikita , J. Montoya , A. Doyle , C. Kirk , A. Vojvodic , H. Y. Hwang , J. K. Norskov , T. F. Jaramillo , Science 2016, 353, 1011. 27701108 10.1126/science.aaf5050

[32] C. Liang , R. R. Rao , K. L. Svane , J. H. L. Hadden , B. Moss , S. B. Scott , M. Sachs , J. Murawski , A. M. Frandsen , D. J. Riley , M. P. Ryan , J. Rossmeisl , J. R. Durrant , I. E. L. Stephens , Nat. Catal. 2024, 7, 763.

[33] K. M. K. Yap , A. Aitbekova , M. Salazar , T. A. Kistler , M. Rodríguez Pabón , M. P. Su , N. B. Watkins , S.‐W. Lee , P. Agbo , A. Z. Weber , J. C. Peters , T. Agapie , A. C. Nielander , H. A. Atwater , T. F. Jaramillo , A. T. Bell , ACS Energy Lett. 2024, 9, 4369.

[34] R. Dronskowski , P. E. Bloechl , J. Phys. Chem. 1993, 97, 8617.

[35] G. Henkelman , A. Arnaldsson , H. Jónsson , Comput. Mater. Sci. 2006, 36, 354.

[36] M. Yu , D. R. Trinkle , J. Chem. Phys. 2011, 134, 064111. 21322665 10.1063/1.3553716

[37] M. Alducin , D. Sánchez‐Portal , A. Arnau , N. Lorente , Phys. Rev. Lett. 2010, 104, 136101. 20481895 10.1103/PhysRevLett.104.136101

[38] M. M. Montemore , M. A. Van Spronsen , R. J. Madix , C. M. Friend , Chem. Rev. 2018, 118, 2816. 29116787 10.1021/acs.chemrev.7b00217

[39] B. M. Comer , N. Bothra , J. R. Lunger , F. Abild‐Pedersen , M. Bajdich , K. T. Winther , ACS Catal. 2024, 14, 5286.

[40] J. Rogal , K. Reuter , M. Scheffler , Phys. Rev. B 2004, 69, 075421.

[41] M. R. Zoric , P. Basera , L. D. Palmer , A. Aitbekova , N. Powers‐Riggs , H. Lim , W. Hu , A. T. Garcia‐Esparza , H. Sarker , F. Abild‐Pedersen , H. A. Atwater , S. K. Cushing , M. Bajdich , A. A. Cordones , ACS Nano 2024, 18, 19538. 39037113 10.1021/acsnano.4c02088 PMC11295187

[42] Z. Zhao , P. Schlexer Lamoureux , A. Kulkarni , M. Bajdich , ChemCatChem 2019, 11, 3423.

[43] I. C. Man , H. Su , F. Calle‐Vallejo , H. A. Hansen , J. I. Martínez , N. G. Inoglu , J. Kitchin , T. F. Jaramillo , J. K. Nørskov , J. Rossmeisl , ChemCatChem 2011, 3, 1159.

[44] M. García‐Mota , M. Bajdich , V. Viswanathan , A. Vojvodic , A. T. Bell , J. K. Nørskov , J. Phys. Chem. C 2012, 116, 21077.

[45] I. Sokolović , M. Reticcioli , M. Čalkovský , M. Wagner , M. Schmid , C. Franchini , U. Diebold , M. Setvín , Proc. Natl. Acad. Sci. USA 2020, 117, 14827. 32527857 10.1073/pnas.1922452117 PMC7334520

[46] T. Pu , A. Setiawan , A. C. Foucher , M. Guo , J.‐M. Jehng , M. Zhu , M. E. Ford , E. A. Stach , S. Rangarajan , I. E. Wachs , ACS Catal. 2024, 14, 406. 38205022 10.1021/acscatal.3c04361 PMC10775145

[47] V. Petrykin , K. Macounova , O. A. Shlyakhtin , P. Krtil , Angew. Chem., Int. Ed. 2010, 49, 4813. 10.1002/anie.200907128 20514655

[48] Z. Shi , J. Li , Y. Wang , S. Liu , J. Zhu , J. Yang , X. Wang , J. Ni , Z. Jiang , L. Zhang , Y. Wang , C. Liu , W. Xing , J. Ge , Nat. Commun. 2023, 14, 843. 36792586 10.1038/s41467-023-36380-9 PMC9932065

[49] Y. Wen , P. Chen , L. Wang , S. Li , Z. Wang , J. Abed , X. Mao , Y. Min , C. T. Dinh , P. D. Luna , R. Huang , L. Zhang , L. Wang , L. Wang , R. J. Nielsen , H. Li , T. Zhuang , C. Ke , O. Voznyy , Y. Hu , Y. Li , W. A. Goddard III , B. Zhang , H. Peng , E. H. Sargent , J. Am. Chem. Soc. 2021, 143, 6482. 33891414 10.1021/jacs.1c00384

[50] N. Bothra , A. Cho , B. Comer , K. Winther , M. Bajdich , ChemRxiv. 2025, 10.26434/chemrxiv-2025-67l8k.

[51] J. K. Nørskov , J. Rossmeisl , A. Logadottir , L. Lindqvist , J. R. Kitchin , T. Bligaard , H. Jónsson , J. Phys. Chem. B 2004, 108, 17886. 39682080 10.1021/jp047349j

[52] J. Rossmeisl , Z.‐W. Qu , H. Zhu , G.‐J. Kroes , J. K. Nørskov , J. Electroanal. Chem. 2007, 607, 83.

[53] V. I. Anisimov , J. Zaanen , O. K. Andersen , Phys. Rev. B 1991, 44, 943. 10.1103/physrevb.44.943 9999600

[54] V. I. Anisimov , F. Aryasetiawan , A. I. Lichtenstein , J. Phys. Condens. Matter 1997, 9, 767.

[55] J. P. Perdew , Y. Wang , Phys. Rev. B 1992, 45, 13244. 10.1103/physrevb.45.13244 10001404

[56] S. R. Bahn , K. W. Jacobsen , Comput. Sci. Eng. 2002, 4, 56.

[57] J. P. Perdew , K. Burke , M. Ernzerhof , Phys. Rev. Lett. 1996, 77, 3865. 10062328 10.1103/PhysRevLett.77.3865

[58] A. Hjorth Larsen , J. Jørgen Mortensen , J. Blomqvist , I. E. Castelli , R. Christensen , M. Dułak , J. Friis , M. N. Groves , B. Hammer , C. Hargus , E. D. Hermes , P. C. Jennings , P. Bjerre Jensen , J. Kermode , J. R. Kitchin , E. Leonhard Kolsbjerg , J. Kubal , K. Kaasbjerg , S. Lysgaard , J. Bergmann Maronsson , T. Maxson , T. Olsen , L. Pastewka , A. Peterson , C. Rostgaard , J. Schiøtz , O. Schütt , M. Strange , K. S. Thygesen , T. Vegge , et al., J. Phys. Condens. Matter 2017, 29, 273002. 28323250 10.1088/1361-648X/aa680e

[59] L. Wang , T. Maxisch , G. Ceder , Phys. Rev. B 2006, 73, 195107. 10.1103/PhysRevLett.97.155704 17155339

[60] Z. Zeng , M. K. Y. Chan , Z.‐J. Zhao , J. Kubal , D. Fan , J. Greeley , J. Phys. Chem. C 2015, 119, 18177.

[61] K. T. Winther , M. J. Hoffmann , J. R. Boes , O. Mamun , M. Bajdich , T. Bligaard , Sci. Data 2019, 6, 75. 31138816 10.1038/s41597-019-0081-y PMC6538711

[62] N. M. Marković , P. N. R. Jr , Surf. Sci. Rep. 2002, 45, 117.

[63] M. Ko , Y. Kim , J. Woo , B. Lee , R. Mehrotra , P. Sharma , J. Kim , S. W. Hwang , H. Y. Jeong , H. Lim , S. H. Joo , J.‐W. Jang , J. H. Kwak , Nat. Catal. 2021, 5, 37.

[64] B. Martindale , Nat. Catal. 2024, 7, 1054.

[65] G. Assat , J.‐M. Tarascon , Nat. Energy 2018, 3, 373.

[66] C. F. Dickens , J. H. Montoya , A. R. Kulkarni , M. Bajdich , J. K. Nørskov , Surf. Sci. 2019, 681, 122.

