# Supplementary material for: Crossing the Oxo‐Peroxo Wall for Selective Electrochemical Epoxidation
Source: Adv Sci (Weinh). 2025 Nov 4;13(4):e17229. doi: 10.1002/advs.202517229 (PMC12822400; doi:10.1002/advs.202517229)
Supplement: Supplementary file 1 — Supporting Information [file ADVS-13-e17229-s001.docx]

**Supporting Information: Crossing the Oxo-Peroxo Wall for Selective Electrochemical Epoxidation**

Pooja Basera^1,2^, Shyama Charan Mandal, Frank Abild-Pedersen, and Michal Bajdich*^2^

1. Department of Chemical Engineering, Stanford University, Stanford, California 94305, United States
2. SUNCAT Center for Interface Science and Catalysis and Liquid Sunlight Alliance, SLAC National Accelerator Laboratory, Menlo Park, California 94025, United States

*E-mail: [bajdich@slac.stanford.edu](mailto:bajdich@slac.stanford.edu)

**Theoretical methods**

**I. OER volcano**

For the OER volcano in main manuscript, the proposed reaction mechanism consists of four consecutive proton and electron transfer steps.

H₂O + * → OH* + (H⁺ + e⁻) (1)

OH* → O* + (H⁺ + e⁻) (2)

O* + H₂O → OOH* + (H⁺ + e⁻) (3)

OOH* → * + O₂(g) + (H⁺ + e⁻) (4)

Considering the OER intermediates to be HO*, O*, and HOO*, free energy diagrams have been constructed, and the oxygen evolution activity has been estimated using OH* formation (Fig 3b) and O*-OH* (SI) free energies as a descriptor. The changes in reaction free energy for the OER are defined as follows: ΔG_1_= ΔG_OH_, ΔG_2_= ΔG_O_ - ΔG_OH_, ΔG_3_= ΔG_OOH_ – ΔG_O_, ΔG_4_= 4.92 – ΔG_OOH_. The analysis performed for the free energies is at standard conditions (pH = 0, T = 298.15 K) and U = 0. We applied the OER oxo scaling relation from Fig3a, given by ΔG_O_ = 1.64ΔG_OH_ + 0.91, along with the universal scaling law for OOH formation^[1]^ ΔG_OOH_=ΔG_OH_ + 3.2 eV. These relationships yield a volcano-shaped correlation between catalytic activity and the calculated OH* Gibbs free energy. Surfaces that bind O*/OH* too strongly, the potential is limited by the formation of OOH* species, whereas for surfaces that bind oxygen too weakly, the potential is limited by the oxidation of OH*. The catalytic performance is further characterized by the magnitude of the potential-determining step for the OER, G^OER^, defined as:

$$G^{\mathrm{OER}}=max\left[ \Delta G_{1},\Delta G_{2},\Delta G_{3},\Delta G_{4} \right]$$

The theoretical overpotential, which is independent of pH, at standard conditions is given by:

$$\eta^{\mathrm{OER}}=\left( G^{\mathrm{OER}}/e \right)-1.23V$$

The shaded blue region in the plot represents the OER-active region determined by the intersection of ΔG_3_ and ΔG_2_ (Fig3b) and is marked with squares indicating the OER-active species.

**II. Hubbard U values**

Table S1: Hubbard U values used for bulk and surface DFT calculations for 3d, 4d, and 5d transition metal oxides. Values are adapted from Ref.^[2–4]^

| Element | Hubbard U value |
| --- | --- |
| Ti | 3.00^[3]^ |
| V | 3.25^[2]^ |
| Cr | 3.5^[3]^ |
| Mn | 3.75^[3]^ |
| Fe | 4.3^[5]^ |
| Co | 3.32 |
| Ni | 6.45^[2]^ |
| Cu | 3.0^[3]^ |
| Mo | 4.38^[4]^ |
| W | 3.0^[4]^ |
| Ce | 4.50^[4]^ |

**III. Components of free energies**.

Table S2: Experimental Gibbs free formation energies and entropic contributions under standard conditions (T=298 K and P=1 bar) as listed in CRC Handbook^[6]^. The calculated DFT total energies are for single molecule in the gas phase. The entropy corrections for the adsorbents on the surface are considered zero, since the main contribution to the entropy is due to the translational entropy. For slab calculation, we use the entropy of gas-phase water calculated at 0.035 bar because this is the equilibrium pressure at T=298 K. This means that the free energy of gas-phase water at these conditions is equal to the free energy of liquid water. The partial pressure is defined as *P=C/k_H_*​, where *C* is propylene concentration, taken as 0.01 M and *k_H_*​ is Henry’s constant. Henry’s constant for alkene (propylene), epoxide (propylene oxide), ketone (Propan-2-one), and aldehyde (propanal) are listed in the table, taken from Ref.^[7]^

|  | $\boldsymbol{\Delta g}_{\mathbf{H2O}}^{\mathbf{ᵉˣᵖ}}$ | E^DFT^ | ZPE | C_v_ | TΔSᵉˣᵖ | *k_H_*  (mol/m^3^Pa) | ZPE - TΔS |
| --- | --- | --- | --- | --- | --- | --- | --- |
| H_2_O(l) | -2.46 (l) | -14.238 | 0.56 |  | 0.216 |  |  |
|  |  |  | 0.56 | 0.103 | 0.675  (0.035bar) |  | -0.012 |
| H_2_(g) | - | -6.774 | 0.268 | 0.090 | 0.408  (1 bar) |  | -0.049 |
| O* | - | - | 0.064 | 0.034 | 0.06 |  | 0.001 |
| O*O*_latt._* |  |  |  |  |  |  | 0.009 |
| OH* | - | - | 0.376 | 0.042 | 0.066 |  | 0.339 |
| OOH* | - | - | 0.471 | 0.077 | 0.134 |  | 0.3637 |
| Alkene |  | -48.689 | 2.098 | 0.138 | 0.801 | 4.7x10^-5^ | 1.435 |
| Epoxide |  | -54.847 | 2.250 | 0.148 | 1.036 | 0.14 | 1.362 |
| Ketone |  | -55.989 | 2.203 | 0.170 | 1.108 | 0.27 | 1.266 |
| Aldehyde |  | -55.647 | 2.224 | 0.164 | 1.086 | 0.099 | 1.301 |

**IV. Vibration corrections for Peroxo (O*O*_latt._*) and Active oxygen (O*) in Peroxo species**

Table S3: The free energies corrections for Peroxo (O*O*_latt._*) and Active oxygen (O*) in Peroxo species. All values presented are in units of eV.

| Surface | (O*O*_latt._*) | |  | O*_latt._* | | O*=(O*O*_latt._*) $\boldsymbol{-}$O*_latt._* |
| --- | --- | --- | --- | --- | --- | --- |
|  | ZPE_1_ | TS_1_ | ZPE_1_-TS_1_ | ZPE_2_ | TS_2_ | (ZPE_1_$-$ TS_1_)$-$ (ZPE_2_$-$TS_2_) |
| Sc_2_O_3_ | 0.147 | 0.106 | 0.041 | 0.083 | 0.04 | -0.002 |
| TiO_2_ | 0.169 | 0.081 | 0.088 | 0.095 | 0.03 | 0.023 |
| CrO_2_ | 0.185 | 0.07 | 0.115 | 0.111 | 0.019 | 0.023 |
| MnO_2_ | 0.162 | 0.108 | 0.054 | 0.071 | 0.0176 | 0.0006 |
| FeO_2_ | 0.15 | 0.109 | 0.041 | 0.09 | 0.033 | -0.016 |
| Fe_2_O_3_ | 0.157 | 0.089 | 0.068 | 0.08 | 0.04 | 0.028 |
| Co_2_O_3_ | 0.167 | 0.086 | 0.081 | 0.092 | 0.032 | 0.021 |
| CuO | 0.144 | 0.106 | 0.038 | 0.083 | 0.037 | -0.008 |
| ZrO_2_ | 0.161 | 0.087 | 0.074 | 0.096 | 0.027 | 0.005 |
| PdO_2_ | 0.171 | 0.082 | 0.089 | 0.098 | 0.025 | 0.016 |
| HfO_2_ | 0.16 | 0.092 | 0.068 | 0.088 | 0.032 | 0.012 |
|  | The average correction for (O*O*_latt._*) and O* is 0.068 eV and 0.0093 eV, respectively. | | | | | |

**V. Surface Pourbaix diagram for PdO (100) and PdO (101) surface**

The surface Pourbaix diagrams were constructed using the Gibbs free energies of H_2_O, H_2_, *OOH, *OH, and *O intermediates on the RHE scale, following previous methods^[8]^. The Gibbs free energies for each step along the oxidation pathway were computed using the computational hydrogen electrode method^[9]^.

**Figure S1.** Surface Pourbaix diagrams for (a) PdO (100) and (b) PdO (101), showing the most-stable surfaces at anodic potentials. The colors correspond to various H, O, and OH coverages, which are labeled in the insets. For the PdO (100) surface, the clean surface is thermodynamically stable, while for PdO (101), the 50% OH-covered surface is stable.


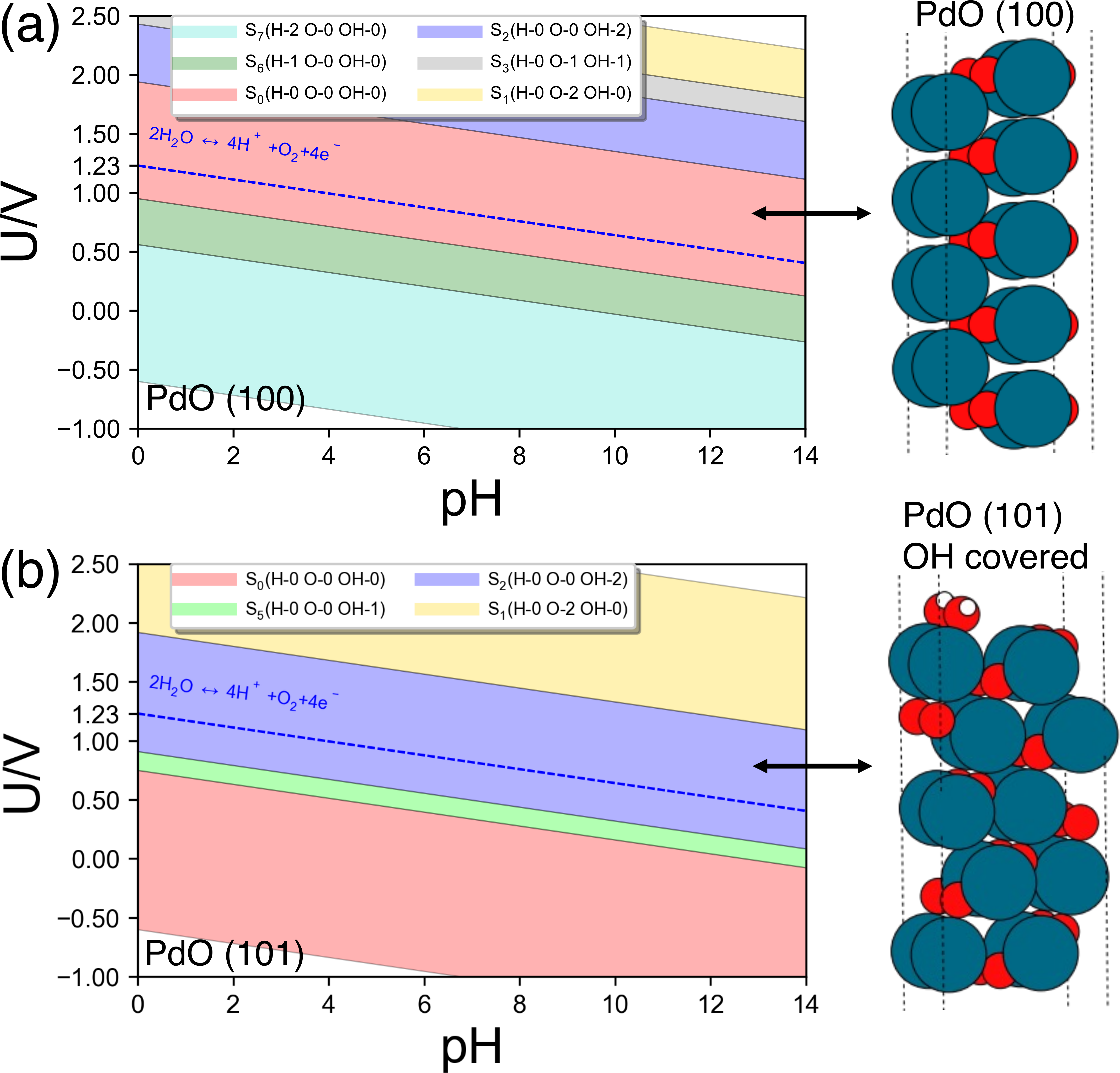


**VI. Reaction free energy diagram from alkene to epoxide, aldehyde and ketone in solution phase.**

**Figure S2.** Gibbs free energy diagram for the conversion of alkene (proylene) to the epoxide (propylene oxide), ketone (Propan-2-one), and aldehyde (propanal) in the solution phase, computed using partial pressure *P=C/k_H_*​, where C=0.01 M is propylene concentration, with Henry’s constant *k_H_*​ of 4.7x10^-5^ , 0.14, 0.27, 0.099 for alkene, epoxide, ketone, and aldehyde, respectively. All of these are two-electron oxidation processes; therefore, the equilibrium potential (E_0_) is calculated by dividing by 2. The E_0_ values for epoxidation, ketone, and aldehyde formation are 0.60 V, $-$0.02 V, and 0.17 V respectively.


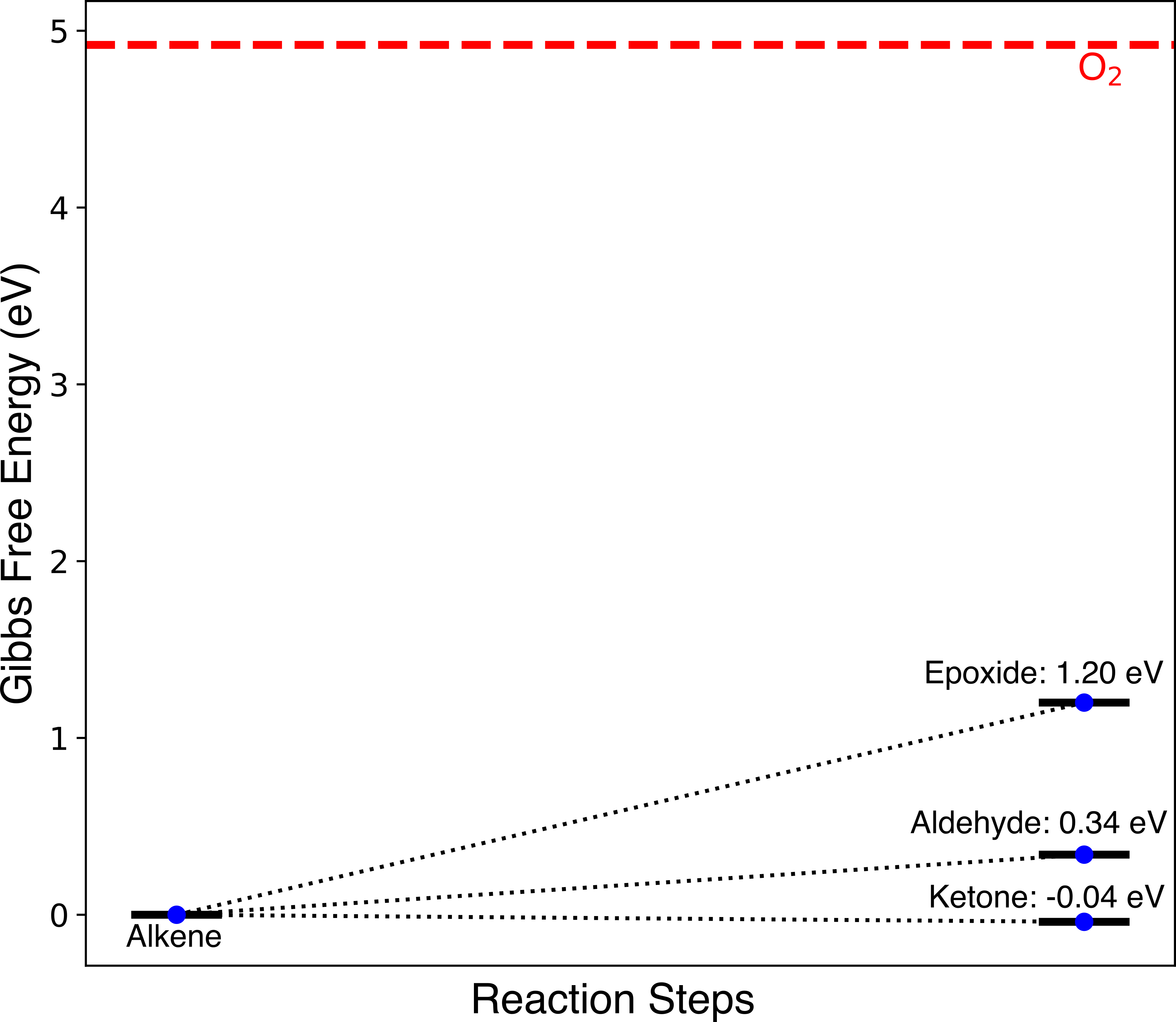


**VII. 2D heat map for OER**


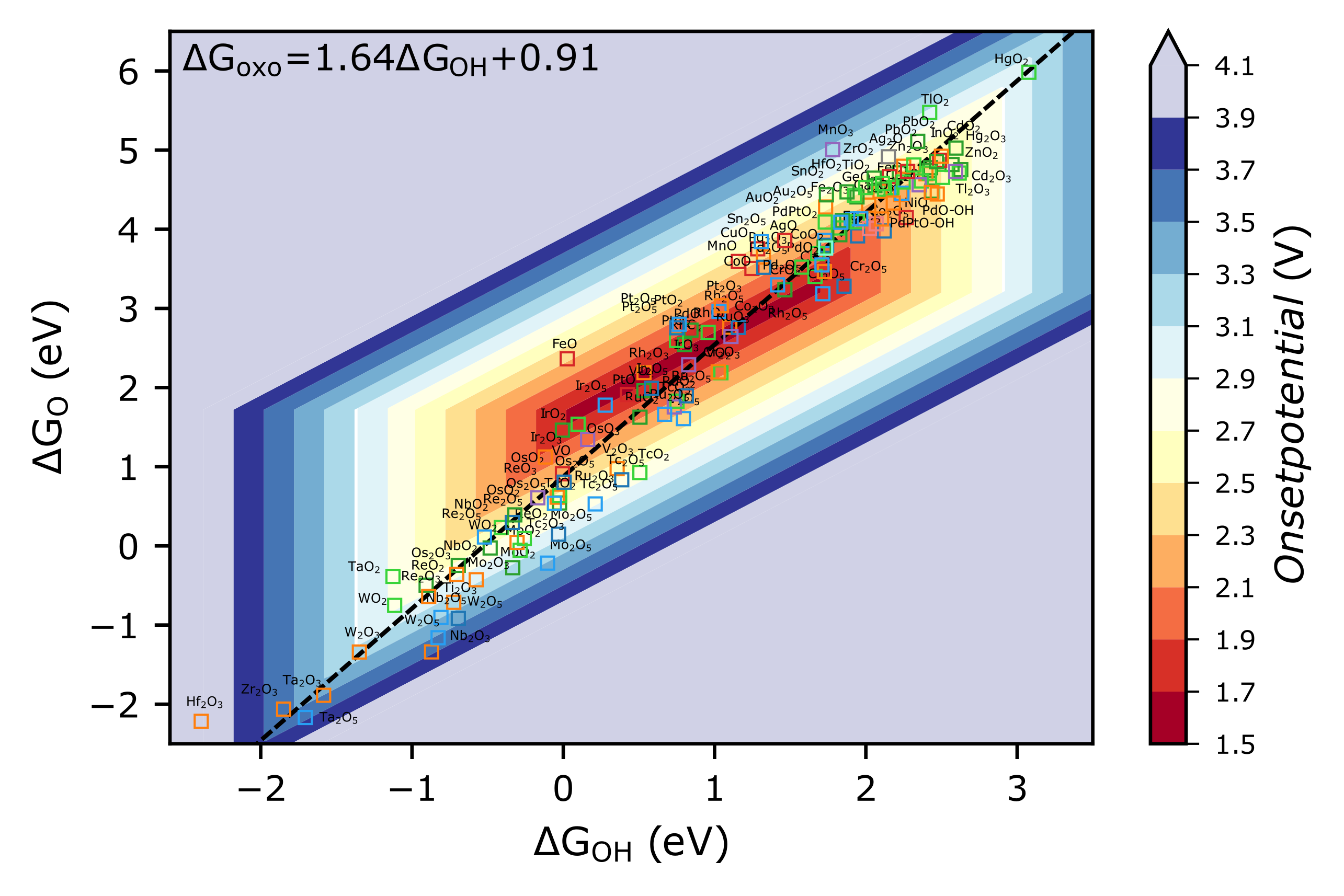


**Figure S3.** OER activity volcano plot shown as a 2D heat map of onset potentials based on Gibbs free energy calculations for $\Delta$G_O_=$\Delta$G_O*_ (oxo, y-axis) and OH* (x-axis) intermediates. The black dashed line indicates the scaling relation for oxo species, and empty symbols denote oxo-active species. For oxo formation, and OER to occur, a minimum onset potential of 1.5 V is required (dark-red region).

**VIII. 2D heat map for Peroxo**


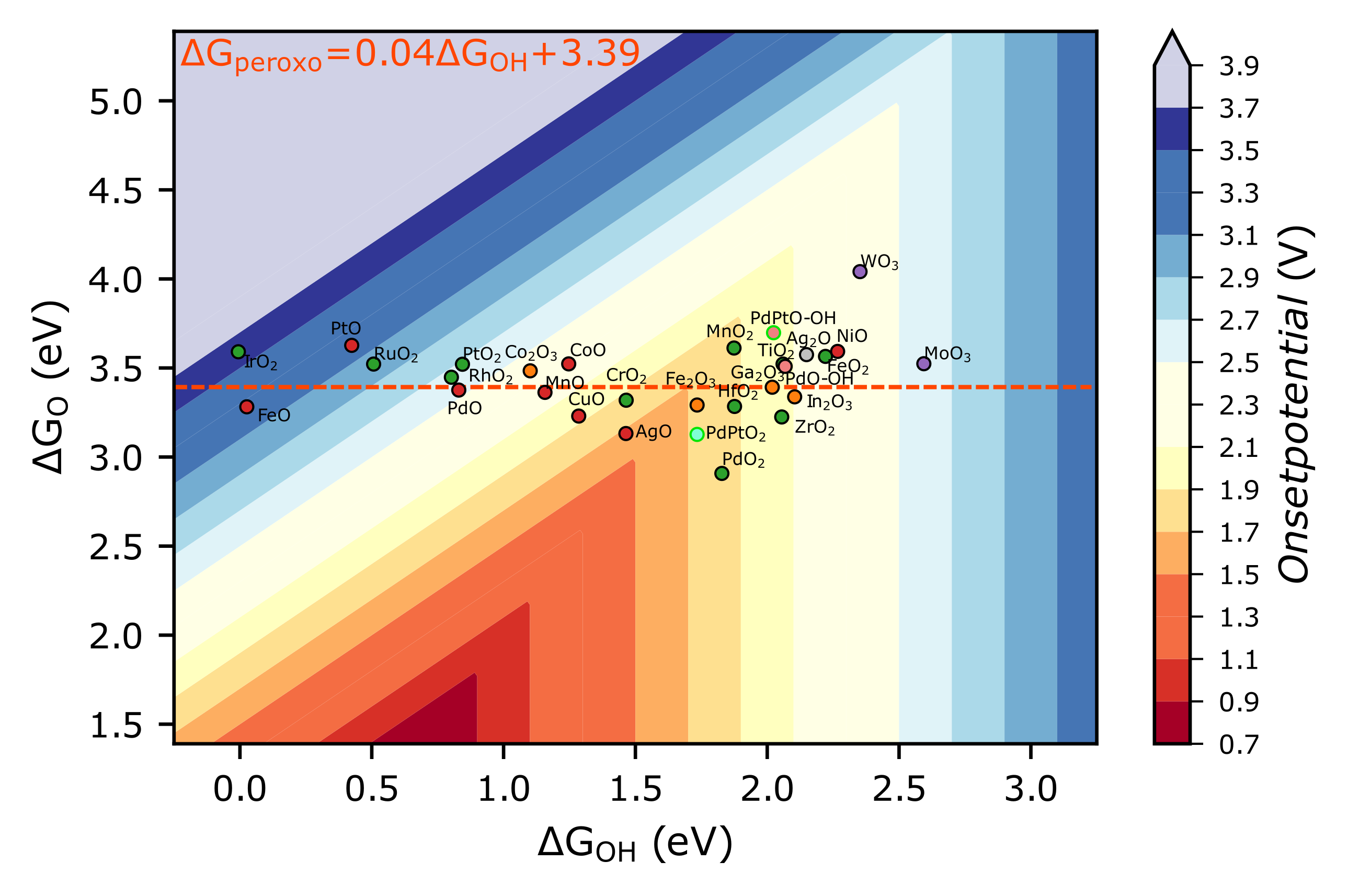


**Figure S4.** Peroxo activity volcano plot shown as a 2D heat map of onset potentials based on Gibbs free energy calculations for $\Delta$G_O_=$\Delta$G_O*O_ (peroxo, y-axis) and OH* (x-axis) intermediates. The red dashed line indicates the scaling relation for peroxo species, and filled symbols denote peroxo-active species. For peroxo formation, and epoxidation to occur, a minimum onset potential of 1.7 V is required, which corresponds to regions of higher onset potentials (yellow regions).

**IX. 1D OER volcano plot with** $\boldsymbol{\Delta}$**G_O_** $\boldsymbol{-}\boldsymbol{\Delta}$**G_OH_ as a descriptor**


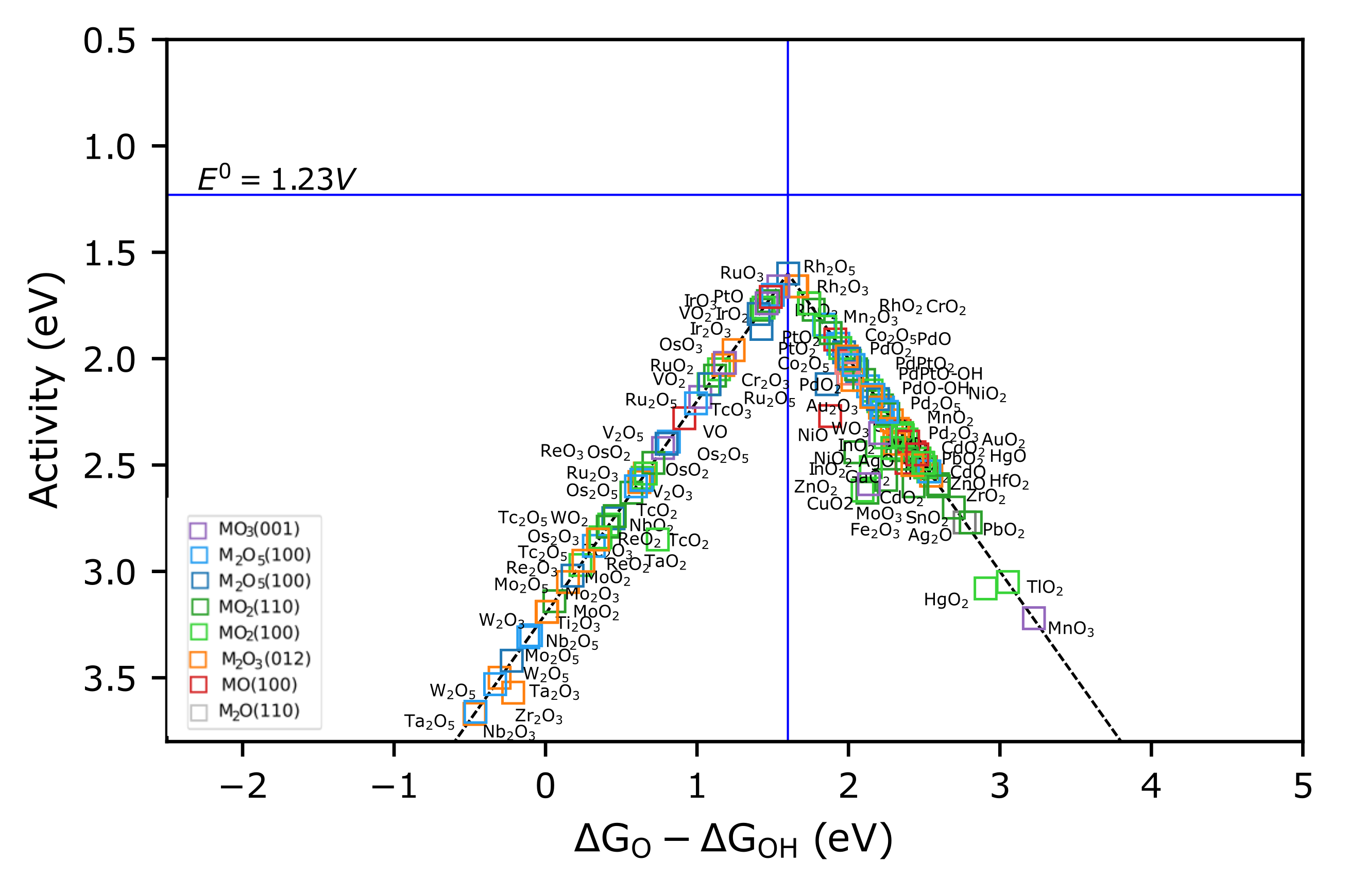


**Figure S5.** OER activity plot: Volcano plot for the OER onset potential. The plot was constructed using the scaling relation shown in Fig3a, based on oxo ($\Delta$G_O_=$\Delta$G_O*_) and OH adsorption energies, with $\Delta$G_O_ $-$ $\Delta$G_OH_ used as a descriptor.

**X. 1D Peroxo volcano plot with** $\boldsymbol{\Delta}$**G_O_** $\boldsymbol{-}\boldsymbol{\Delta}$**G_OH_ as a descriptor**


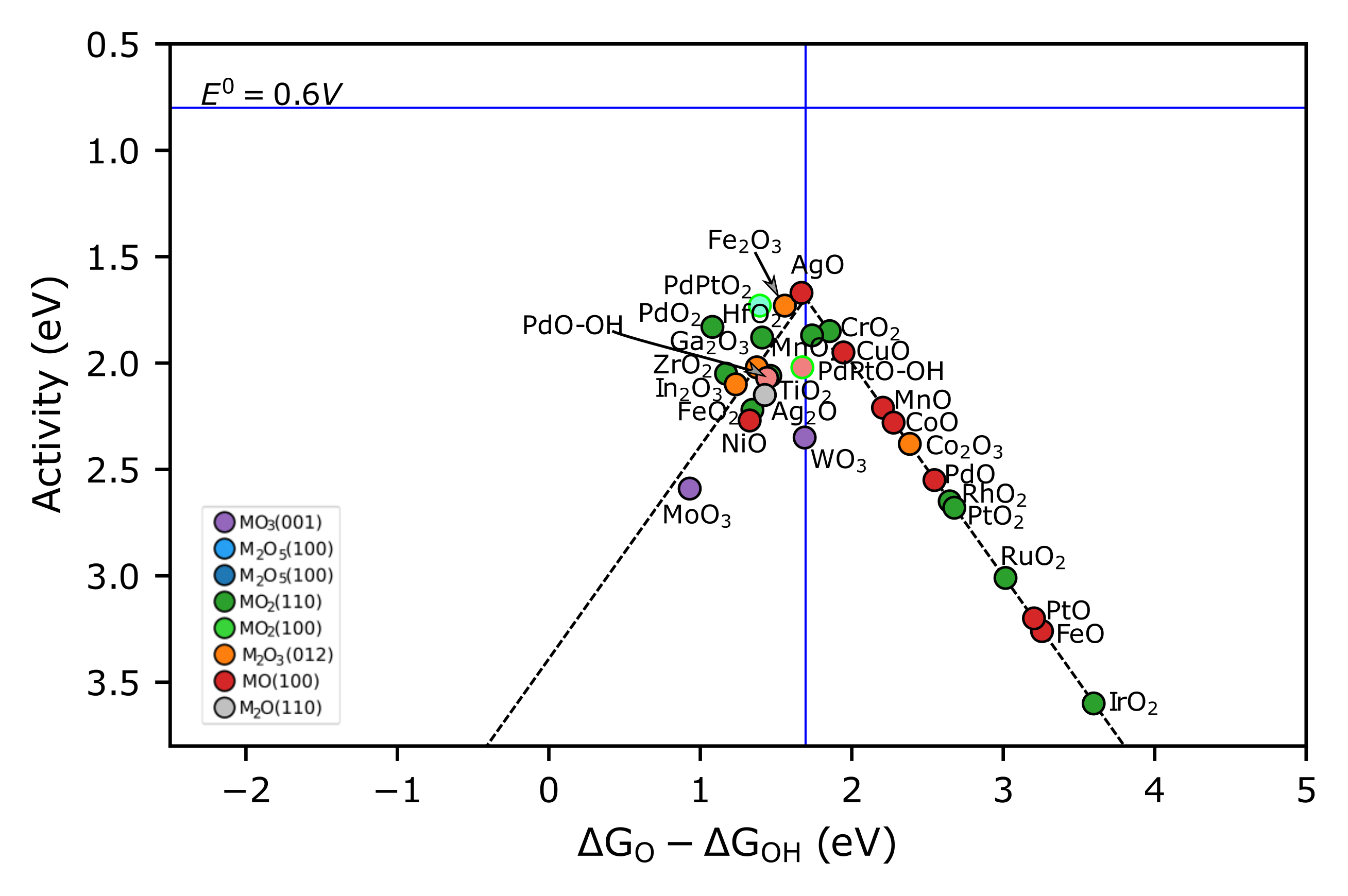


**Figure S6.** Peroxo activity plot: Volcano plot for the Peroxo onset potential. The plot was constructed using the scaling relation shown in Fig3a, based on peroxo ($\Delta$G_O_=$\Delta$G_O*O_) and OH adsorption energies, with $\Delta$G_O_ $-$ $\Delta$G_OH_ used as a descriptor.

**XI. Oxo-Peroxo Wall for 3d, 4d, and 5d metal oxides**

**Figure S7.** Oxygen adsorption energies across the Oxo-Peroxo Wall for transition metal-oxides. Same plot as shown in main manuscript Fig2, but all additional cases included here, such as Sc_2_O_3_, Y_2_O_3_, La_2_O_3_, and Au_2_O_3_. Dark blue and light blue correspond to 1.0 and 0.5 monolayer (ML) O- atom coverage, respectively.

**XII. Magnetic moments for oxo (O*), oxyl (M–O•), peroxo (O*O), and superoxo (O₂*) species on various surfaces**

**Figure S8.** The magnetic moments (μ_B_) for oxo (O*), oxyl (M–O·), peroxo (O*O), and superoxo (O₂*) species on various surfaces plotted in Bohr magnetons (µ_B_). Moments are reported on the O atom for oxo/oxyl and as the sum over the O–O*_latt._* pair for peroxo/superoxo. Squares denote oxo/oxyl species (oxo: RuO₂; oxyl: TiO₂, ZrO₂, HfO₂, FeO₂, Ga₂O₃ etc.). Filled circles denote peroxo/superoxo species. Peroxo is typically diamagnetic with data points fall on the grey dashed line at ≈0 μ_B_, though certain high spin 3d oxides exhibit small finite moments. For superoxo, MoO_3_ and AgO show O–O moments of ≈0.8 and ≈0.4 μ_B_, consistent with a doublet and a weak-doublet (near-singlet) state, respectively. As a guide: oxo (M=O²⁻) ≈0–0.2 μ_B_ (on O); oxyl (M–O·) ≈0.6–1.2 μ_B_ (on O); peroxo (O₂²⁻) ≈0–0.3 μ_B_ (O–O*_latt._* pair); superoxo (O₂⁻) ≈0.6–1.2 μ_B_ (O–O*_latt._*  pair); triplet O₂ ≈1.6–2.4 μB.


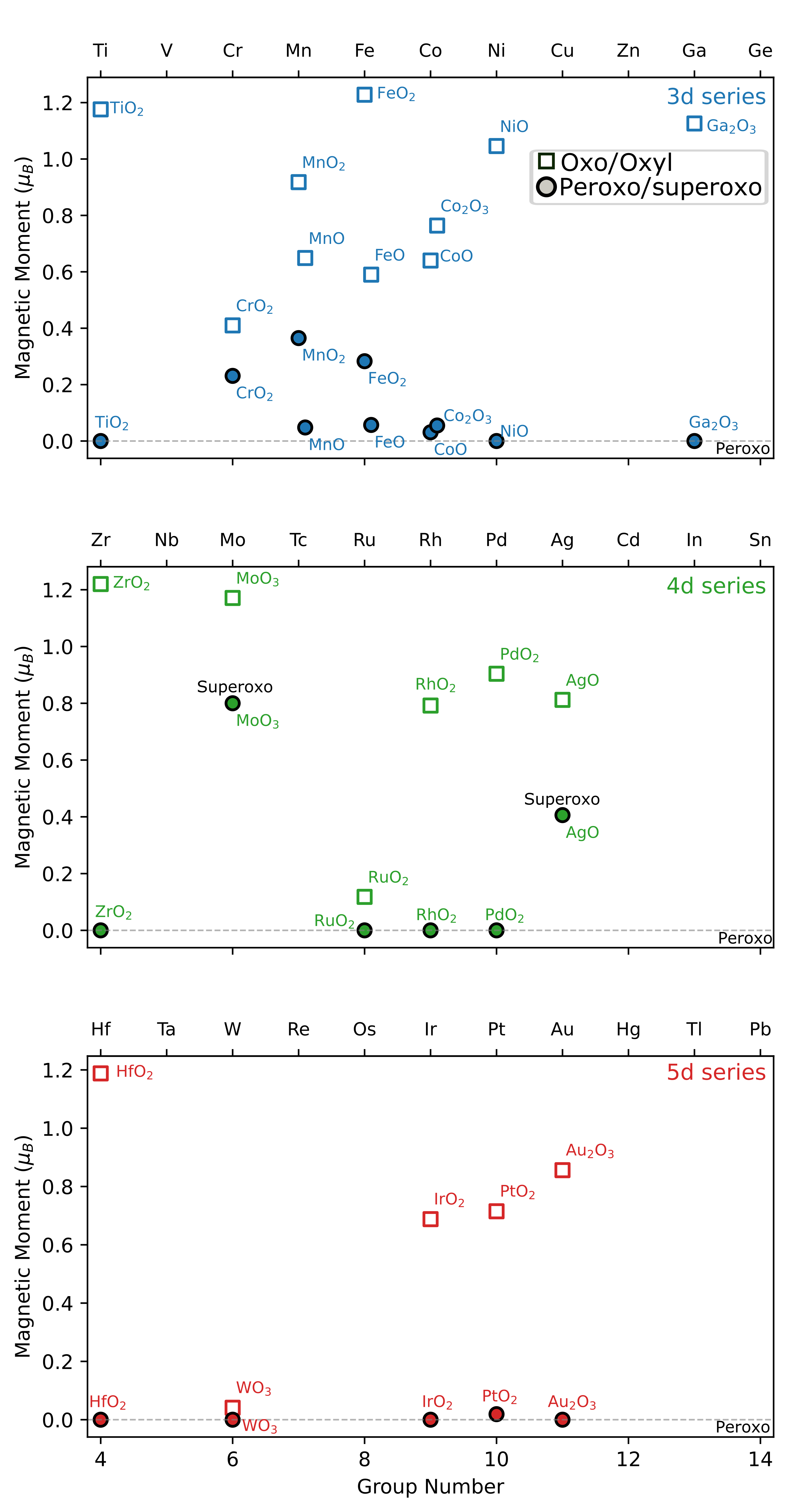


**XIII. Molecular orbital diagram for 5d transition metal oxides (TMO)**

**Figure S9.** Molecular orbital (MO) diagram for an octahedrally coordinated *5d* transition metal oxide (TMO) (e.g. Iridium, Ir) bound to six oxygen ligands. The MOs are hybridized and have character arising from both the transition metal and ligand atomic orbitals is adapted from References^10,11^ .

In transition-metal oxides (TMO), the ligand environment around the metal is non-spherical, so the metal d levels split according to site symmetry. Two effects govern this splitting: (i) electrostatic crystal-field repulsion and (ii) *d-p* hybridization with oxygen *2p* orbitals. For an octahedrally coordinated compound (as in IrO₂), the d levels of the TM ion split into doubly degenerate *e_g_* (pointing at ligands) and triply degenerate *t_2g_* (between ligands) subspaces. This separation is called crystal field splitting. The *t_2g_* levels lie therefore at lower energies than the *e_g_* states. The filling up of orbitals depends on the competition between the crystal field splitting and the Hund’s rule coupling.

To explicitly connect to surfaces, we consider IrO_2_ as an example drawn from Refs.^[10,11]^. In the case of IrO_2_, the large spatial extent of Ir *5d* orbitals gives rise to stronger *d-p* interaction and larger splitting between the *t_2g_* and *e_g_* levels as compared to the splitting in *3d* TMO. In an IrO_6_ octahedron (Ir^4+^ = 5d^5^), the six O^2-^ ligands (6×2*p*^6^ = 36 e⁻) form symmetry-adapted ligand orbitals i.e. σ (*a_1g_, e_g_, t_1u_*), π (*t_2g_, t_1u_*), and nonbonding (*t_1g_, t_2u_*). Mixing these with Ir *5d/6s/6p* yields M–O molecular orbitals in which strong σ overlap produces a large *e_g_(σ)/e_g_(σ*)* split, while weaker *π* overlap gives a smaller *t_2g_(π)/t_2g_(π*)* split. In the strong-field limit typical of *5d* oxides, the O-based bonding/NB levels are filled by the 36 O *2p* electrons, and the five Ir *d* electrons occupy the metal-centered *t_2g_(π*)*, i.e. (*t_2g_(π*)⁵*) while *e_g_(σ*)* remains empty (*e_g_(σ*)⁰*), i.e., low-spin *d^5^* = *t_2g_^5^e_g_^0^*. This occupancy keeps M–O *σ* strong (*σ** unfilled) but weakens M–O *π* (*π** occupied). In case of PdO_2_ (*Pd^4+^, 4d^6^*), adding an electron into the metal–oxygen *π** further destabilizes the M–O *π* bonding. As a result, O–O coupling is favored and peroxo/superoxo preferred over oxo species. The molecular orbital diagram is adapted from references^[10,11]^.

**Tabulated adsorption energies**

**Table S4**.The adsorption energies obtained for OH*, O*, OO* adsorption for metal oxide surfaces. The data and structures are stored on Catalysis Hub^[12]^ at [https://www.catalysis-hub.org/publications/Basera](https://www.catalysis-hub.org/publications/Basera%20Revising%202024)Implications2025

| Surface Composition | Facet (site) | Equation | Reaction Energy |
| --- | --- | --- | --- |
| HfO_2_-rutile | 100 | H_2_O(g) - H_2_(g) + * $\to$ O* | 4.42943 |
| HfO_2_-rutile | 100 | H_2_O(g) - 0.5H_2_(g) + * $\to$ OH* | 1.5856 |
| HfO_2_-rutile | 110 | H_2_O(g) - H_2_(g) + * $\to$ O* | 4.47125 |
| HfO_2_-rutile | 110 (peroxo) | H_2_O(g) - H_2_(g) + * $\to$ O* | 3.27505 |
| HfO_2_-rutile | 110 | H_2_O(g) - 0.5H_2_(g) + * $\to$ OH* | 1.53663 |
| MnO-Fm3m | 100 (peroxo) | 2H_2_O(g) - 2H_2_(g) + * $\to$ 2O* | 6.70933 |
| MnO-Fm3m | 100 | 2H_2_O(g) - 2H_2_(g) + * $\to$ 2O* | 7.18527 |
| MnO-Fm3m | 100 | 2H_2_O(g) - H_2_(g) + * $\to$ 2OH* | 1.97492 |
| PdO_2_-rutile | 100 | H_2_O(g) - H_2_(g) + * $\to$ O* | 3.52041 |
| PdO_2_-rutile | 100 | H_2_O(g) - 0.5H_2_(g) + * $\to$ OH* | 1.23628 |
| PdO_2_-rutile | 110-lc | H_2_O(g) - H_2_(g) + * $\to$ O* | 3.93053 |
| PdO_2_-rutile | 110-lc (peroxo) | H_2_O(g) - H_2_(g) + * $\to$ O* | 2.85456 |
| PdO_2_-rutile | 110-lc (epoxy) | C_3_H_6_(g) + H_2_O(g) - H_2_(g) + * $\to$ C_3_H_6_O* | 1.73814 |
| PdO_2_-rutile | 110-lc | H_2_O(g) - 0.5H_2_(g) + * $\to$ OH* | 1.48839 |
| PdO_2_-rutile | 110-lc | 2.0H_2_O(g) - 1.5H_2_(g) + *$\to$HO2* | 4.44613 |
| PdO_2_-rutile | 110-lc  (O-vac) | -1.0H_2_O(g) + H_2_(g) + O_2_(g) + * $\to$ O* | -3.64213 |
| PdO_2_-rutile | 110 | H_2_O(g) - H_2_(g) + * $\to$ O* | 3.87379 |
| PdO_2_-rutile | 110 (peroxo) | H_2_O(g) - H_2_(g) + * $\to$ O* | 2.95422 |
| PdO_2_-rutile | 110 | H_2_O(g) - 0.5H_2_(g) + * $\to$ OH* | 0.99558 |
| ZrO_2_-rutile | 100 | H_2_O(g) - H_2_(g) + * $\to$ O* | 4.57736 |
| ZrO_2_-rutile | 100 | H_2_O(g) - 0.5H_2_(g) + * $\to$ OH* | 1.77081 |
| ZrO_2_-rutile | 110 | H_2_O(g) - H_2_(g) + * $\to$ O* | 4.64453 |
| ZrO_2_-rutile | 110 (peroxo) | H_2_O(g) - H_2_(g) + * $\to$ O* | 3.21616 |
| ZrO_2_-rutile | 110 | H_2_O(g) - 0.5H_2_(g) + * $\to$ OH* | 1.71593 |
| CoO-Fm3m | 100 (peroxo) | 2H_2_O(g) - 2H_2_(g) + * $\to$ 2O* | 7.02948 |
| CoO-Fm3m | 100 | 2H_2_O(g) - 2H_2_(g) + * $\to$ 2O* | 7.01098 |
| CoO-Fm3m | 100 | 2H_2_O(g) - H_2_(g) + * $\to$ 2OH* | 2.1551 |
| In_2_O_3_-corundum | 012 | H_2_O(g) - H_2_(g) + * $\to$ O* | 4.12888 |
| In_2_O_3_-corundum | 012 (peroxo) | H_2_O(g) - H_2_(g) + * $\to$ O* | 3.32978 |
| In_2_O_3_-corundum | 012 | H_2_O(g) - 0.5H_2_(g) + * $\to$ OH* | 1.76468 |
| FeO_2_-rutile | 100 | H_2_O(g) - H_2_(g) + * $\to$ O* | 4.526 |
| FeO_2_-rutile | 100 | H_2_O(g) - 0.5H_2_(g) + * $\to$ OH* | 1.83325 |
| FeO_2_-rutile | 110 | H_2_O(g) - H_2_(g) + * $\to$ O* | 4.7116 |
| FeO_2_-rutile | 110 (peroxo) | H_2_O(g) - H_2_(g) + * $\to$ O* | 3.55689 |
| FeO_2_-rutile | 110 | H_2_O(g) - 0.5H_2_(g) + * $\to$ OH* | 1.88222 |
| FeO-Fm3m | 100 (peroxo) | 2H_2_O(g) - 2H_2_(g) + * $\to$ 2O* | 6.54638 |
| FeO-Fm3m | 100 | 2H_2_O(g) - 2H_2_(g) + * $\to$ 2O* | 4.72498 |
| FeO-Fm3m | 100 | 2H_2_O(g) - H_2_(g) + * $\to$ 2OH* | -0.627403 |
| RuO_2_-rutile | 100 | H_2_O(g) - H_2_(g) + * $\to$ O* | 1.82744 |
| RuO_2_-rutile | 100 | H_2_O(g) - 0.5H_2_(g) + * $\to$ OH* | 0.412744 |
| RuO_2_-rutile | 110 | H_2_O(g) - H_2_(g) + * $\to$ O* | 1.62545 |
| RuO_2_-rutile | 110 (peroxo) | H_2_O(g) - H_2_(g) + * $\to$ O* | 3.50397 |
| RuO_2_-rutile | 110 | H_2_O(g) - 0.5H_2_(g) + * $\to$ OH* | 0.168 |
| PdPt | 101 | H_2_O(g) - H_2_(g) + * $\to$ O* | 4.00936 |
| PdPt | 101 (peroxo) | H_2_O(g) - H_2_(g) + * $\to$ O* | 3.68998 |
| PdPt | 101 | H_2_O(g) - 0.5H_2_(g) + * $\to$ OH* | 1.68516 |
| PtO_2_-rutile | 100 | H_2_O(g) - H_2_(g) + * $\to$ O* | 2.59434 |
| PtO_2_-rutile | 100 | H_2_O(g) - 0.5H_2_(g) + * $\to$ OH* | 0.409204 |
| PtO_2_-rutile | 110 | H_2_O(g) - H_2_(g) + * $\to$ O* | 2.72525 |
| PtO_2_-rutile | 110 (peroxo) | H_2_O(g) - H_2_(g) + * $\to$ O* | 3.51093 |
| PtO_2_-rutile | 110 | H_2_O(g) - 0.5H_2_(g) + * $\to$ OH* | 0.504258 |
| PtO_2_-rutile | 110-lc | H_2_O(g) - H_2_(g) + * $\to$ O* | 2.89575 |
| PtO_2_-rutile | 110-lc (peroxo) | H_2_O(g) - H_2_(g) + * $\to$ O* | 3.33149 |
| PtO_2_-rutile | 110-lc | 2.0H_2_O(g) - 1.5H_2_(g) + *$\to$HO2* | 3.83838 |
| PtO_2_-rutile | 110-lc | H_2_O(g) - 0.5H_2_(g) + * $\to$ OH* | 0.705704 |
| PtO_2_-rutile | 110-lc | C_3_H_6_(g) + H_2_O(g) - H_2_(g) + * $\to$ C_3_H_6_O* | -0.123209 |
| PtO_2_-rutile | 110-lc  (O-vac) | -1.0H_2_O(g) + H_2_(g) + O_2_(g) + * $\to$ O* | -4.78206 |
| CuO | 111 | H_2_O(g) - H_2_(g) + * $\to$ O* | 3.75258 |
| CuO | 111 (peroxo) | H_2_O(g) - H_2_(g) + * $\to$ O* | 3.22177 |
| CuO | 111 | H_2_O(g) - 0.5H_2_(g) + * $\to$ OH* | 0.946088 |
| Mo_16_O_51_ | 001 | H_2_O(g) - H_2_(g) + * $\to$ O* | 4.72875 |
| Mo_16_O_51_ | 001 (peroxo) | H_2_O(g) - H_2_(g) + * $\to$ O* | 3.51526 |
| Mo_16_O_51_ | 001 | H_2_O(g) - 0.5H_2_(g) + * $\to$ OH* | 2.25524 |
| NiO-Fm3m | 100 (peroxo) | 2H_2_O(g) - 2H_2_(g) + * $\to$ 2O* | 7.16929 |
| NiO-Fm3m | 100 | 2H_2_O(g) - 2H_2_(g) + * $\to$ 2O* | 8.29081 |
| NiO-Fm3m | 100 | 2H_2_O(g) - H_2_(g) + * $\to$ 2OH* | 3.85488 |
| WO_3_ | 001 | H_2_O(g) - H_2_(g) + * $\to$ O* | 4.56049 |
| WO_3_ | 001 (peroxo) | H_2_O(g) - H_2_(g) + * $\to$ O* | 4.03221 |
| WO_3_ | 001 | H_2_O(g) - 0.5H_2_(g) + * $\to$ OH* | 2.00322 |
| CrO_2_-rutile | 100 | H_2_O(g) - H_2_(g) + * $\to$ O* | 3.40305 |
| CrO_2_-rutile | 100 | H_2_O(g) - 0.5H_2_(g) + * $\to$ OH* | 1.3257 |
| CrO_2_-rutile | 110 | H_2_O(g) - H_2_(g) + * $\to$ O* | 3.23404 |
| CrO_2_-rutile | 110 (peroxo) | H_2_O(g) - H_2_(g) + * $\to$ O* | 3.31058 |
| CrO_2_-rutile | 110 | H_2_O(g) - 0.5H_2_(g) + * $\to$ OH* | 1.12565 |
| Fe_2_O_3_-corundum | 012 | H_2_O(g) - H_2_(g) + * $\to$ O* | 4.28078 |
| Fe_2_O_3_-corundum | 012 (peroxo) | H_2_O(g) - H_2_(g) + * $\to$ O* | 3.28376 |
| Fe_2_O_3_-corundum | 012 | H_2_O(g) - 0.5H_2_(g) + * $\to$ OH* | 1.39469 |
| Ga_2_O_3_-corundum | 012 | H_2_O(g) - H_2_(g) + * $\to$ O* | 4.30423 |
| Ga_2_O_3_-corundum | 012 (peroxo) | H_2_O(g) - H_2_(g) + * $\to$ O* | 3.38349 |
| Ga_2_O_3_-corundum | 012 | H_2_O(g) - 0.5H_2_(g) + * $\to$ OH* | 1.67997 |
| Nb_2_O_5_-C12c | 100-lc | H_2_O(g) - H_2_(g) + * $\to$ O* | -0.905427 |
| Nb_2_O_5_-C12c | 100-lc | H_2_O(g) - 0.5H_2_(g) + * $\to$ OH* | -1.14766 |
| Nb_2_O_5_-C12c | 100-hc | H_2_O(g) - H_2_(g) + * $\to$ O* | 3.24652 |
| Nb_2_O_5_-C12c | 100-hc (peroxo) | H_2_O(g) - H_2_(g) + * $\to$ O* | 3.11398 |
| Nb_2_O_5_-C12c | 100-hc | H_2_O(g) - 0.5H_2_(g) + * $\to$ OH* | 0.256407 |
| TiO_2_-rutile | 100 | H_2_O(g) - H_2_(g) + * $\to$ O* | 4.50349 |
| TiO_2_-rutile | 100 | H_2_O(g) - 0.5H_2_(g) + * $\to$ OH* | 1.82797 |
| TiO_2_-rutile | 110 | H_2_O(g) - H_2_(g) + * $\to$ O* | 4.54936 |
| TiO_2_-rutile | 110 (peroxo) | H_2_O(g) - H_2_(g) + * $\to$ O* | 3.51361 |
| TiO_2_-rutile | 110 | H_2_O(g) - 0.5H_2_(g) + * $\to$ OH* | 1.7209 |
| Ti_2_O_3_-corundum | 012 | H_2_O(g) - H_2_(g) + * $\to$ O* | -0.713285 |
| Ti_2_O_3_-corundum | 012 | H_2_O(g) - 0.5H_2_(g) + * $\to$ OH* | -1.06336 |
| Ag_2_O | 110 | H_2_O(g) - H_2_(g) + * $\to$ O* | 4.90392 |
| Ag_2_O | 110 (peroxo) | H_2_O(g) - H_2_(g) + * $\to$ O* | 3.57222 |
| Ag_2_O | 110 | H_2_O(g) - 0.5H_2_(g) + * $\to$ OH* | 1.81249 |
| Co_2_O_3_-corundum | 012 | H_2_O(g) - H_2_(g) + * $\to$ O* | 2.76214 |
| Co_2_O_3_-corundum | 012 (peroxo) | H_2_O(g) - H_2_(g) + * $\to$ O* | 3.47554 |
| Co_2_O_3_-corundum | 012 | H_2_O(g) - 0.5H_2_(g) + * $\to$ OH* | 0.762122 |
| V_2_O_5_-C12c | 100-lc | H_2_O(g) - H_2_(g) + * $\to$ O* | 1.60696 |
| V_2_O_5_-C12c | 100-lc | H_2_O(g) - 0.5H_2_(g) + * $\to$ OH* | 0.45486 |
| V_2_O_5_-C12c | 100-hc | H_2_O(g) - H_2_(g) + * $\to$ O* | 3.9347 |
| V_2_O_5_-C12c | 100-hc (peroxo) | H_2_O(g) - H_2_(g) + * $\to$ O* | 2.98641 |
| V_2_O_5_-C12c | 100-hc | H_2_O(g) - 0.5H_2_(g) + * $\to$ OH* | 1.44212 |
| RhO_2_-rutile | 100 | H_2_O(g) - H_2_(g) + * $\to$ O* | 2.69526 |
| RhO_2_-rutile | 100 | H_2_O(g) - 0.5H_2_(g) + * $\to$ OH* | 0.618568 |
| RhO_2_-rutile | 110 | H_2_O(g) - H_2_(g) + * $\to$ O* | 2.54177 |
| RhO_2_-rutile | 110 (peroxo) | H_2_O(g) - H_2_(g) + * $\to$ O* | 3.43988 |
| RhO_2_-rutile | 110 | H_2_O(g) - 0.5H_2_(g) + * $\to$ OH* | 0.462479 |
| Y_2_O_3_-corundum | 012 | H_2_O(g) - H_2_(g) + * $\to$ O* | 4.37661 |
| Y_2_O_3_-corundum | 012 (peroxo) | H_2_O(g) - H_2_(g) + * $\to$ O* | 2.8335 |
| Y_2_O_3_-corundum | 012 | H_2_O(g) - 0.5H_2_(g) + * $\to$ OH* | 1.33638 |
| IrO_2_-rutile | 100 | H_2_O(g) - H_2_(g) + * $\to$ O* | 1.53642 |
| IrO_2_-rutile | 100 | H_2_O(g) - 0.5H_2_(g) + * $\to$ OH* | -0.241255 |
| IrO_2_-rutile | 110 | H_2_O(g) - H_2_(g) + * $\to$ O* | 1.46179 |
| IrO_2_-rutile | 110 (peroxo) | H_2_O(g) - H_2_(g) + * $\to$ O* | 3.58265 |
| IrO_2_-rutile | 110 | H_2_O(g) - 0.5H_2_(g) + * $\to$ OH* | -0.344821 |
| PdO | 101 | H_2_O(g) - H_2_(g) + * $\to$ O* | 2.74331 |
| PdO | 101 (peroxo) | H_2_O(g) - H_2_(g) + * $\to$ O* | 3.36848 |
| PdO | 101 | H_2_O(g) - 0.5H_2_(g) + * $\to$ OH* | 0.488409 |
| La_2_O_3_-corundum | 012 | H_2_O(g) - H_2_(g) + * $\to$ O* | 4.32849 |
| La_2_O_3_-corundum | 012 (peroxo) | H_2_O(g) - H_2_(g) + * $\to$ O* | 2.52393 |
| La_2_O_3_-corundum | 012 | H_2_O(g) - 0.5H_2_(g) + * $\to$ OH* | 1.37377 |
| Sc_2_O_3_-corundum | 012 | H_2_O(g) - H_2_(g) + * $\to$ O* | 4.51056 |
| Sc_2_O_3_-corundum | 012 (peroxo) | H_2_O(g) - H_2_(g) + * $\to$ O* | 3.0447 |
| Sc_2_O_3_-corundum | 012 | H_2_O(g) - 0.5H_2_(g) + * $\to$ OH* | 1.60349 |
| PtO | 101 | H_2_O(g) - H_2_(g) + * $\to$ O* | 1.91026 |
| PtO | 101 (peroxo) | H_2_O(g) - H_2_(g) + * $\to$ O* | 3.62076 |
| PtO | 101 | H_2_O(g) - 0.5H_2_(g) + * $\to$ OH* | 0.0837388 |
| TiO-Fm3m | 100 | 2H_2_O(g) - 2H_2_(g) + * $\to$ 2O* | 0.207247 |
| TiO-Fm3m | 100 | 2H_2_O(g) - H_2_(g) + * $\to$ 2OH* | -2.52855 |
| AgO | 111 | H_2_O(g) - H_2_(g) + * $\to$ O* | 3.85804 |
| AgO | 111 (peroxo) | H_2_O(g) - H_2_(g) + * $\to$ O* | 3.12541 |
| AgO | 111 | H_2_O(g) - 0.5H_2_(g) + * $\to$ OH* | 1.12588 |
| MnO_2_-rutile | 100 | H_2_O(g) - H_2_(g) + * $\to$ O* | 4.01555 |
| MnO_2_-rutile | 100 | H_2_O(g) - 0.5H_2_(g) + * $\to$ OH* | 1.47976 |
| MnO_2_-rutile | 110 | H_2_O(g) - H_2_(g) + * $\to$ O* | 4.09478 |
| MnO_2_-rutile | 110 (peroxo) | H_2_O(g) - H_2_(g) + * $\to$ O* | 3.6031 |
| MnO_2_-rutile | 110 | H_2_O(g) - 0.5H_2_(g) + * $\to$ OH* | 1.5347 |
| PdO-OH-covered-square-planar | 101 | H_2_O(g) - H_2_(g) + * $\to$ O* | 4.06861 |
| PdO-OH-covered-square-planar | 101 (peroxo) | H_2_O(g) - H_2_(g) + * $\to$ O* | 3.50006 |
| PdO-OH-covered-square-planar | 101 | H_2_O(g) - 0.5H_2_(g) + * $\to$ OH* | 1.72903 |
| PdPt | 110-lc | H_2_O(g) - H_2_(g) + * $\to$ O* | 3.76284 |
| PdPt | 110-lc (peroxo) | H_2_O(g) - H_2_(g) + * $\to$ O* | 3.11817 |
| PdPt | 110-lc | H_2_O(g) - 0.5H_2_(g) + * $\to$ OH* | 1.39456 |
| PdPt | 110-lc | 2.0H_2_O(g) - 1.5H_2_(g) + *$\to$HO2* | 3.601 |
| PdPt | 110-lc | H_2_O(g) - 0.5H_2_(g) + * $\to$ OH* | 1.394 |
| PdPt | 110-lc  (epoxy) | C_3_H_6_(g) + H_2_O(g) - H_2_(g) + * $\to$ C_3_H_6_O* | 0.5299 |
| PdPt | 110-lc  (O-vac) | -1.0H_2_O(g) + H_2_(g) + O_2_(g) + * $\to$ O* | -3.9909 |
| Au_2_O_3_ | 100 | H_2_O(g) - H_2_(g) + * $\to$ O* | 3.16382 |
| Au_2_O_3_ | 100 | H_2_O(g) - H_2_(g) + * $\to$ O* | 2.86809 |
| Au_2_O_3_ | 100 | H_2_O(g) - 0.5H_2_(g) + * $\to$ OH* | 0.71471 |

# **REFERENCES**

[1] I. C. Man, H. Su, F. Calle‐Vallejo, H. A. Hansen, J. I. Martínez, N. G. Inoglu, J. Kitchin, T. F. Jaramillo, J. K. Nørskov, J. Rossmeisl, *ChemCatChem* **2011**, *3*, 1159.

[2] A. Jain, G. Hautier, S. P. Ong, C. J. Moore, C. C. Fischer, K. A. Persson, G. Ceder, *Phys. Rev. B* **2011**, *84*, 045115.

[3] Z. Zhao, P. Schlexer Lamoureux, A. Kulkarni, M. Bajdich, *ChemCatChem* **2019**, *11*, 3423.

[4] B. M. Comer, N. Bothra, J. R. Lunger, F. Abild-Pedersen, M. Bajdich, K. T. Winther, *ACS Catal.* **2024**, *14*, 5286.

[5] A. J. Tkalych, H. L. Zhuang, E. A. Carter, *ACS Catal.* **2017**, *7*, 5329.

[6] *CRC Handbook of Chemistry and Physics, 93rd Ed.; CRC Press: Boca Raton, FL, 2011-2012*, **n.d.**

[7] R. Sander, *Atmospheric Chem. Phys.* **2015**, *15*, 4399.

[8] Y. Liu, L. Guo, *J. Chem. Phys.* **2020**, *152*, 100901.

[9] J. K. Nørskov, J. Rossmeisl, A. Logadottir, L. Lindqvist, J. R. Kitchin, T. Bligaard, H. Jónsson, *J. Phys. Chem. B* **2004**, *108*, 17886.

[10] S. Chander, S. K. Tripathi, *Mater. Adv.* **2022**, *3*, 7198.

[11] V. M. Katukuri, *PhD Thesis Tech. Univ. Dresd.* **2014**.

[12] K. T. Winther, M. J. Hoffmann, J. R. Boes, O. Mamun, M. Bajdich, T. Bligaard, *Sci. Data* **2019**, *6*, 75.
